# The temporal landscape of recursive splicing during Pol II transcription elongation in human cells

**DOI:** 10.1371/journal.pgen.1007579

**Published:** 2018-08-27

**Authors:** Xiao-Ou Zhang, Yu Fu, Haiwei Mou, Wen Xue, Zhiping Weng

**Affiliations:** 1 Program in Bioinformatics and Integrative Biology, University of Massachusetts Medical School, Worcester, Massachusetts, United States of America; 2 Bioinformatics Program, Boston University, Boston, Massachusetts, United States of America; 3 RNA Therapeutics Institute, University of Massachusetts Medical School, Worcester, Massachusetts, United States of America; 4 Program in Molecular Medicine, University of Massachusetts Medical School, Worcester, Massachusetts, United States of America; 5 Department of Molecular, Cell and Cancer Biology, University of Massachusetts Medical School, Worcester, Massachusetts, United States of America; 6 Department of Biochemistry and Molecular Pharmacology, University of Massachusetts Medical School, Worcester, Massachusetts, United States of America; University of California San Francisco, UNITED STATES

## Abstract

Recursive splicing (RS) is an evolutionarily conserved process of removing long introns via multiple steps of splicing. It was first discovered in *Drosophila* and recently proven to occur also in humans. The detailed mechanism of recursive splicing is not well understood, in particular, whether it is kinetically coupled with transcription. To investigate the dynamic process that underlies recursive splicing, we systematically characterized 342 RS sites in three human cell types using published time-series data that monitored synchronized Pol II elongation and nascent RNA production with 4-thiouridine labeling. We found that half of the RS events occurred post-transcriptionally with long delays. For at least 18–47% RS introns, we detected RS junction reads only after detecting canonical splicing junction reads, supporting the notion that these introns were removed by both recursive splicing and canonical splicing. Furthermore, the choice of which splicing mechanism was used showed cell type specificity. Our results suggest that recursive splicing supplements, rather than replaces, canonical splicing for removing long introns.

## Author summary

Nascent precursor RNAs newly transcribed from a DNA template are processed into mature RNAs by splicing out introns and ligating exons. Recursive splicing (RS) is a special kind of splicing whereby long introns are removed piecewise in multiple consecutive steps. Only nine human introns are known to undergo recursive splicing, and in this paper, we have developed a computational pipeline to search for RS introns in the human transcriptome using labeled and synchronized RNA sequencing data. We identified 342 RS sites in three human cell types and characterized their sequence and epigenetic features. We further show that half of the RS events occur post-transcriptionally and RS introns are removed by both recursive splicing and canonical splicing.

## Introduction

Recursive splicing (RS)—a multi-step process to excise long introns from pre-mRNAs—was first identified in the Ultrabithorax (*Ubx*) gene of *Drosophila melanogaster* [1]. Each recursive splice site (RS site; also called a ratchet site in *Drosophila*) contains a pair of juxtaposed 3′ and 5′ splice sites, dividing a long intron into two segments to be spliced out sequentially. The RS site first functions as a 3′ splice site, pairing with the upstream 5′ splice site to remove the upstream intronic segment. The reconstituted 5′ splice site interacts with the downstream 3′ splice site for the subsequent removal of the remaining intronic segment.

Genome-wide studies revealed that hundreds of long fly introns perform recursive splicing [2, 3] and nine long introns of human neuronal genes contain high-confidence RS sites [4]. RS sites are generally followed by an “RS exon” that is crucial for the recognition of the RS site [3, 4]. The RS exons are retained in low-abundance transcript isoforms, resulting from the competition between the two 5′ splice sites—one at the RS site and the other one at the 3′ end of the RS exon [3, 4]. Some annotated cassette exons could also function as RS exons [4]. These findings bridge the gap between recursive splicing and alternative splicing in humans, suggesting that recursive splicing can, in principle, use the machinery of alternative splicing. However, the features of RS exons that differ from cassette exons have not been thoroughly investigated and the mechanism of conversion between them remains elusive.

Abundant evidence indicates that most human splicing occurs co-transcriptionally, and the transcriptional elongation by RNA polymerase II (Pol II) plays an essential role in regulating splicing by kinetic coupling [5, 6]. Recursive splicing, which is a special case of splicing, may also be regulated co-transcriptionally. Indeed, the Pol II elongation rate has been shown to exert an impact on the efficiency of recursive splicing of the fly *Ubx* gene [7]. However, the extent of coupling between recursive splicing and Pol II elongation in humans has not yet been studied. Moreover, it was proposed that recurrent splicing was constitutive in flies [2], but it is not known whether this is also the case for humans.

Half of the human genes contain at least one intron longer than 10 kb, and 14% of all human introns are over 10 kb (S1A Fig), much more common than fly introns (4%). To obtain a better understanding of the role of recursive splicing in long intron excision, we developed an integrative RS sites identification pipeline and applied it to previously published RNA sequencing datasets with 4-thiouridine (4sU) tagging of nascent RNAs upon synchronization of Pol II elongation with 5,6-dichlorobenzimidazole 1-ß-D-ribofuranoside (DRB) [8]. We identified 342 candidate RS sites in three human cell lines: embryonic carcinoma PA1 cells, embryonic stem cells (H9), and differentiated forebrain neuron progenitor cells. We measured the extent of kinetic coupling between recursive splicing and Pol II elongation and found half of the RS events occur post-transcriptionally with long delays. We also looked for evidence for or against constitutive recursive splicing in these human cells and found that many recursive splicing events co-occurred with canonical splicing. Furthermore, the choice of recursive splicing versus canonical splicing appears to be cell-type specific. Together, our results indicate that recursive splicing is part of the cell’s arsenal for removing long introns.

## Results

### A computational pipeline for annotating recursive splice site in humans

**Fig 1:**
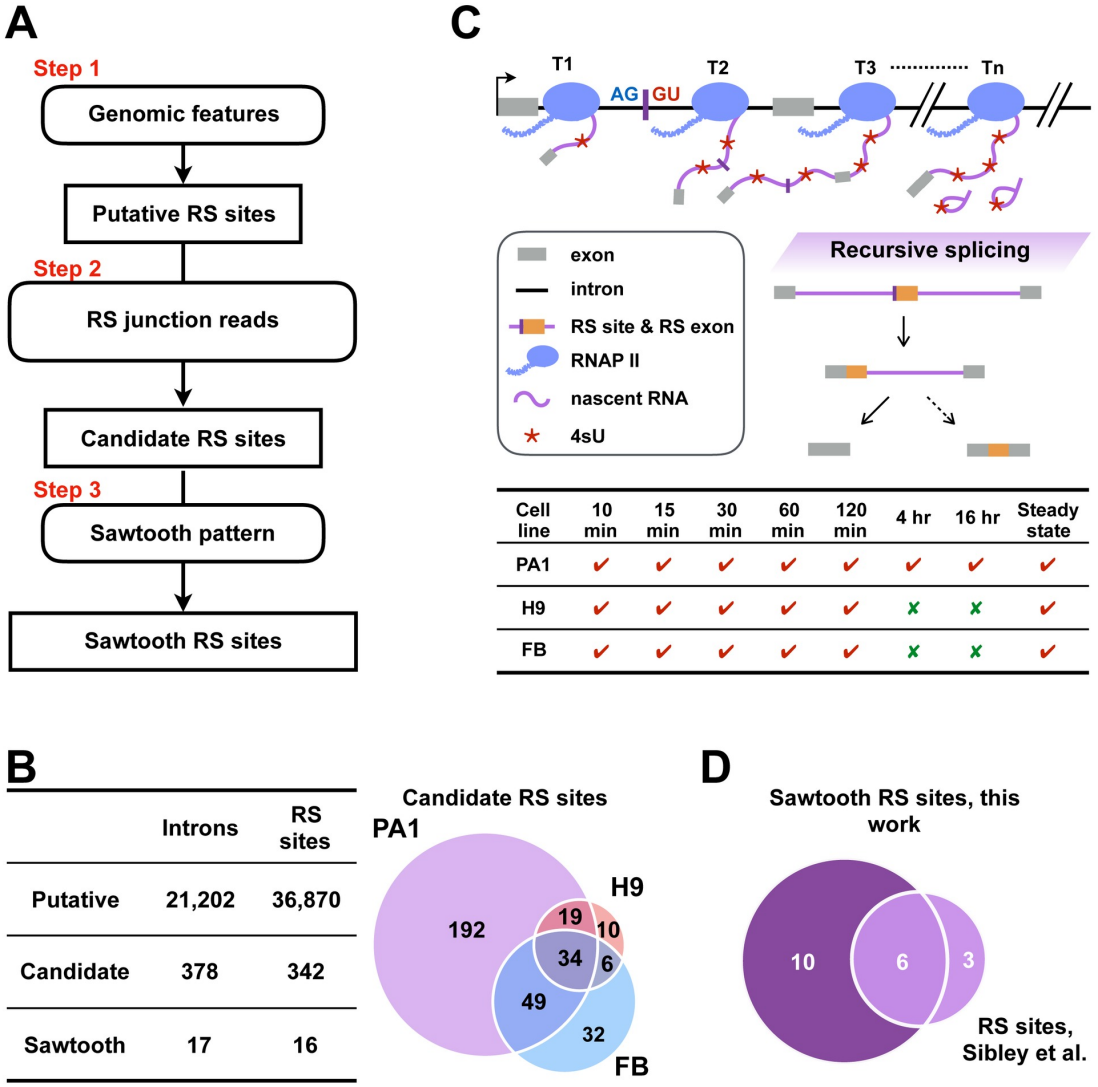
Genome-wide identification of RS sites in humans using 4sUDRB-seq datasets. (A) A computational pipeline for systematically identifying RS sites. The pipeline contains three steps (Materials and methods). Candidate RS sites result after the first two steps, and sawtooth RS sites result after the third step. (B) A table lists the numbers of putative, candidate, and sawtooth RS sites and RS introns identified in three human cell lines (left panel). Hundreds of candidate RS sites were identified in more than two cell lines (right panel). (C) A schematic to illustrate 4sUDRB-seq captures recursive splicing. 4sU-labeled, newly synthesized, total RNAs from PA1, H9, and H9-differentiated FB cells were collected at different time points (T1, T2, …, Tn) after the commence of synchronized Pol II elongation, and transcripts with or without recursive splicing were captured accordingly (top panel). 4sUDRB-seq was performed on PA1 cells at seven time points, but on H9 and FB cells at the first five time points (bottom panel). (D) Among the 16 sawtooth RS sites with the sawtooth pattern, six sites (38%; light purple) were previously validated, and the remaining 10 (62%; dark purple) are new.

RS sites have several genomic features that can facilitate their identification. First, RS sites match the AGGT motif, i.e., the 3′ splice site consensus (AG) immediately followed by the 5′ splice site consensus (GT), and they are evolutionarily conserved [2, 4]. Second, RS sites are enriched in long introns because short introns are too spatially constrained to perform multiple-step splicing [9]. Third, the 3′ splice sites of RS sites need to be sufficiently strong to be recognized by the spliceosome at the first step of recursive splicing [10]. We systematically evaluate these features on the nine reported human RS sites [4] and canonical splice sites (S1A–S1C Fig). The results were then used to set cutoffs in the first step of our RS site identification pipeline (Fig 1A, step 1; S2 Fig, step 1; Materials and methods).

We first identified all potential sites that matched the AGGT motif in long introns. There are 65,019 long introns in the human genome, defined as being longer than 5,000 nucleotides (nt). We then eliminated the sites that overlapped with any splice sites in the UCSC Known Genes annotation, retaining those with strong 3′ splice sites (strength ≥ 5 computed with MaxEntScan [11]) and high evolutionary conservation (PhastCons score ≥ 0.5 [12]). We found 36,870 putative RS sites in 21,202 introns (Fig 1B, left; S1 Table), covering 33% of long introns, with an average of 1.7 putative RS sites per intron.

Another feature that we used to detect RS sites is the splicing intermediate that joins the 3′ splice site of each RS site with the 5′ splice site of the upstream exon. The second step of our pipeline identifies such RS junction reads by generating a set of putative 5′-exon–RS site junctions and realigning all sequencing reads against them. This strategy was used to map RS junctions in flies and increased the specificity of the method [2]. Sites with at least three unique supporting junction reads were identified and designated candidate RS sites for the remaining analyses (Fig 1A, step 2; S2 Fig, step 2; Materials and methods).

The last feature of RS sites implemented in our pipeline is the "sawtooth" pattern in the steady-state total RNA-seq data (with ribosomal RNAs removed). All nine previously reported human RS sites show strong evidence of a sawtooth pattern in the steady-state RNA-seq data in human forebrain cells (S1D Fig). However, the sawtooth pattern is difficult to detect for several reasons. First, the RNA-seq profile is noisy; thus, the sawtooth pattern is only detectable at a sufficiently high sequencing depth for genes that are expressed at high levels. Second, the sawtooth pattern varies by intron length and shows strong tissue specificity [13]. Third, the sawtooth pattern is based on the assumption that recursive splicing is a rapid process [2, 4, 13], and to what extent that is the case has not been evaluated. Consequently, we used the sawtooth pattern as the most stringent criterion in the third step of our pipeline, and candidate RS sites with the sawtooth pattern are called sawtooth RS sites (Fig 1A, step 3; S2 Fig, step 3; Materials and methods).

In summary, we have established a computational pipeline that integrates various known genomic or expression features of recursive splicing and uses stringent thresholding for RS site detection (Fig 1A; S2 Fig; Materials and methods).

### Detection of recursive splicing in humans using 4sUDRB-seq data

The previous study identified recursive splicing in humans using steady-state total RNA-seq [4]. We postulated that time-course 4sUDRB-seq data might be more effective than steady-state RNA-seq data for detecting RS sites because the former could capture splicing intermediates that would exist only for a limited time after transcription. Thus, we analyzed the published 4sUDRB-seq data in human ovarian carcinoma PA1 cells, human H9 embryonic stem cells, and forebrain (FB) neuronal progenitor cells differentiated from H9 cells, previously generated for studying the biogenesis of circular RNAs [8]. Upon transcription synchronization by reversible DRB inhibition, nascent RNAs tagged by 4sU were sequenced at five time points from 10 minutes to 2 hours for all three cell types, and additionally at 4 and 16 hours for PA1 cells (Fig 1C). In addition to providing data for determining Pol II elongation rates genome-wide, 4sUDRB-seq data at such long durations capture complete transcription and splicing of genes with long introns. The sequencing depths of these datasets are particularly high, with the five deepest datasets ranging from 260 to 387 million reads (S3A Fig). These data are particularly suitable for studying recursive splicing.

Using our RS site prediction pipeline, we identified 342 candidate RS sites in PA1, H9, or FB cells (S2 Table), and 108 of them (31.6%) were in two or more cell lines, significantly enriched over random chance (permutation test *p*-value < 1.00×10^−4^; Fig 1B). Of these, 16 RS sites show a significant sawtooth pattern (S3 Table), including six of the nine known human RS sites [4] (Fig 1D). The other three known RS sites also showed the sawtooth pattern (S1D Fig), but we did not detect sufficient junction reads for two sites, and the third site overlapped with an annotated cassette exon and was filtered out by our pipeline. Because we could identify only the 5′ splice site of RS introns that corresponds to each RS site but not the 3′ splice site, we included all potential 3′ splice sites according to gene annotations. As a result, 342 candidate RS sites and 16 sawtooth RS sites correspond to 378 and 17 introns, respectively (Fig 1B).

We identified more RS sites in the FB neuronal cells than in the undifferentiated H9 cells at the 30 and 120 min time points, even after we downsampled the datasets to the same sequencing depth (S3B Fig). This result is consistent with the previous finding that genes that were highly expressed in neurons tended to have long introns [4, 14]. We performed Gene Ontology enrichment analysis on the genes harboring RS sites and found that they were enriched in ontology terms such as neuron projection guidance and axon guidance (S3C Fig), also consistent with previous findings [4].

We asked whether our pipeline could rediscover the 435 putative RS sites by Sibley et al., which were based on the RS site motif (AGGT) and RS junction reads in their total RNA-seq data [4]. S4A Fig shows the breakdown of these 435 sites: 53% of them are in introns longer than 5 kb, 26% of them pass our evolutionary conservation cutoff, and only twenty sites passed all the criteria in Step 1 of our pipeline, indicating that our pipeline is stricter than that of Sibley et al. Among these 20 putative RS sites, twelve also passed Step 2 of our pipeline and were classified as candidate RS sites. We detected the sawtooth pattern for nine of these ten sites, including six sites for which Sibley et al. also detected the sawtooth pattern (red sites in S1D Fig). Thus, among the 36,870 putative RS sites, 342 candidate RS sites, and 16 sawtooth RS sites identified by our pipeline, 36,850 (99.9%) putative RS sites, 330 (96.5%) candidate RS sites, and 7 (43.8%) sawtooth RS sites are reported for the first time.

**Fig 2:**
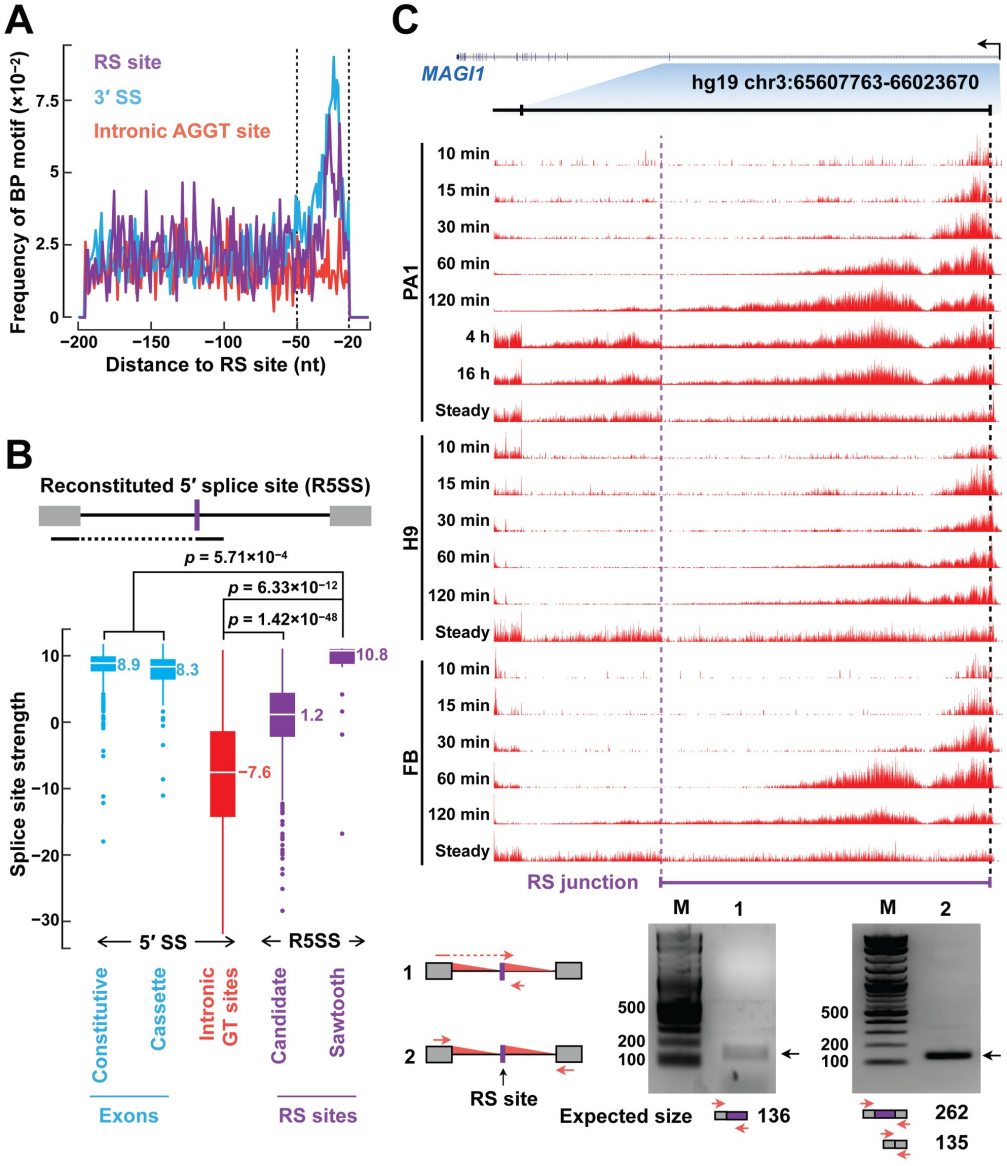
Validation of identified RS sites. (A) The branch-point motif is enriched in the 20–50 bp window upstream of RS sites (purple line) and canonical 3′ splice sites (blue line), but not randomly sampled intronic AGGT sites (red line). (B) RS sites reconstitute strong 5′ splice sites. The 5′ splice sites reconstituted from RS sites are significantly stronger than randomly sampled intronic GT sites. The 5′ splice sites reconstituted from sawtooth RS sites are even stronger than the 5′ splice sites of cassette exons and constitutive exons. Median splice site strengths and Wilcoxon rank-sum test *p*-values are indicated. (C) We validated a novel RS site by RT-PCR. Top, 4sUDRB-seq signal tracks show the sawtooth pattern for an RS site in the first intron of the *MAGI1* gene. Bottom, RT-PCR validation of the RS site. This recursive splicing junction was detected with recursive splicing specific primers (lane 1), but not with the primers that mapped to the exons (lane 2). PCR primers are indicated as red arrows in the figure and their sequences are provided in S6 Table.

We proceeded to computationally validate our candidate RS sites in four ways. First, we asked if our RS sites were supported by junction reads. Searching for junction reads in 806 RNA-seq datasets from the ENCODE project totaling more than 70 billion reads, we found RS junction reads supporting 60% of our putative RS sites and 89% of our candidate RS sites (S4B Fig). Second, we asked whether we could detect enrichment of the branch point motif upstream of the candidate RS sites. Indeed, there is an enrichment for the branch point motif at 20–50 nt upstream the RS sites, but not for randomly selected intronic AGGT sites (Fig 2A). Third, we searched for lariat reads from the two introns flanking each RS site, as such lariat reads provide direct evidence of the recursive splicing (S4C Fig, top panel). Typically, lariat reads are rare, but we did find 266 reads supporting 23 RS intron lariats of 15 RS sites in publicly available RNA-seq data (S4 Table). Nineteen of the 23 identified branch points reside 20–50 nt upstream of the RS sites or their downstream 3′ splice sites, and an A is evident at the branch point (S4C Fig, bottom left panel). We also found 153 lariat reads in these RNA-seq datasets that supported 12 of the 230 putative RS sites in introns (≥ 5k nt) by Sibley et al. (S4C Fig, bottom right panel). Fourth, we asked whether our RS sites could also reconstitute the extended 5′ splice site motif, given that our pipeline only required a strong match to the 3′ splice site motif and the GT dinucleotide in the reconstituted 5′ splice site. Indeed, the reconstituted 5′ splice sites of our candidate RS sites (logo shown in S4D Fig, top) are significantly stronger than the 5′ splice sites of randomly selected intronic sites that start with a GT dinucleotide (median strengths 1.2 vs.−7.6; *p*-value = 1.42×10^−48^), albeit weaker than the 5′ splice sites of cassette exons and constitutive exons (Fig 2B). The reconstituted 5′ splice sites of our sawtooth RS sites (logo shown in S4D Fig, bottom) are slightly stronger than the 5′ splice sites of cassette exons and constitutive exons (*p*-value = 5.71×10^−4^; Fig 2B). These four analyses provide support for the validity of our predicted RS sites.

We performed RT-PCR and Sanger sequencing experiments to validate a novel RS site in the first intron of the *MAGI1* gene, which could be detected in all three cell lines with a clear sawtooth pattern (Fig 2C, top). Using a pair of primers specific for the intermediate product of recursive splicing, we detected a product at the correct size (a 136-bp band in the lane 1 of Fig 2C) and further verified it with Sanger sequencing. This intermediate product was not detected by two primers that annealed to the flanking exons (only a 135-bp band but not a 262-bp band in the lane 2 of Fig 2C).

### 4sUDRB-seq data reveal more RS sites than total RNA-seq data

We compared 4sUDRB-seq with total RNA-seq for their efficacy in finding RS sites. Among the candidate RS sites, we identified in PA1, H9, and FB cells using 4sUDRB-seq data, only 15%, 38%, and 42% could be detected using the steady-state RNA-seq data in the corresponding cell types, which appeared to support our hypothesis that 4sUDRB-seq data were more effective than RNA-seq data for identifying RS sites. However, this result was partly due to that the 4sUDRB-seq datasets totaled more reads than the RNA-seq datasets; thus, we must perform a fair comparison at equal sequencing depth. Given that the Pol II elongation rate is on average 2,520 nt/min, 97% of human genes would have been completely transcribed after 120 mins [8]. Accordingly, we performed detailed comparison by downsampling the 120-min 4sUDRB-seq and steady-state RNA-seq datasets to 80 M reads each, the lowest sequencing depth among all datasets (S3A Fig). Averaged over these three cell lines, we detected roughly the same number of RS sites using the 4sUDRB-seq datasets as using the steady-state RNA-seq datasets (15.3 vs. 14; S5A Fig). We also compared the 4-hr and 16-hr 4sUDRB-seq data with the steady-state RNA-seq data in PA1 cells at the sequencing depth of 80 M. We detected the most RS sites using the 16-hr 4sUDRB-seq data, 1.6 times as many as using the steady-state RNA-seq data (S5B Fig). The majority of the RS junctions detected using the steady-state RNA-seq data were also detected using the 4sUDRB-seq data, and the junctions were supported by comparable numbers of reads in the two types of data (S5C Fig). Taken together, our results indicate that 4sUDRB-seq data with long durations can capture more RS events than steady-state RNA-seq data.

A distinct advantage of time-series 4sUDRB-seq data is that they can provide quantitative information on the temporal progression of recursive splicing. For example, we found three RS sites in the *PDE4D* gene, two of which (RS1 and RS3) were also reported by Sibley et al. All three RS sites show the sawtooth pattern in PA1 cells, but the pattern emerges at different time points (S5D Fig, left). Pol II transcribes from two independent promoters of *PDE4D*, and recursive splicing could be detected at as early as 60 min for the promoter-proximal RS1 and RS2 sites, while only after 120 mins for the promoter-distal RS3. Once the sawtooth pattern starts, it persists throughout later time points, supported by junction reads that correspond to the recursive splicing intermediates at all these time points (S5D Fig, right). The sawtooth pattern is weaker at 16 hr than at 2 hr and 4 hr for most of the sawtooth RS sites in PA1 cells (7 out of 12, including *PDE4D*), suggesting that the optimal time points for detecting the sawtooth pattern are 2 hr and 4 hr.

### Genomic features of human RS sites

To characterize the genomic features of human RS sites identified by our pipeline (Fig 1B), we analyzed intron length, evidence for RS exons, GC content, and evolutionary conservation.

**Fig 3:**
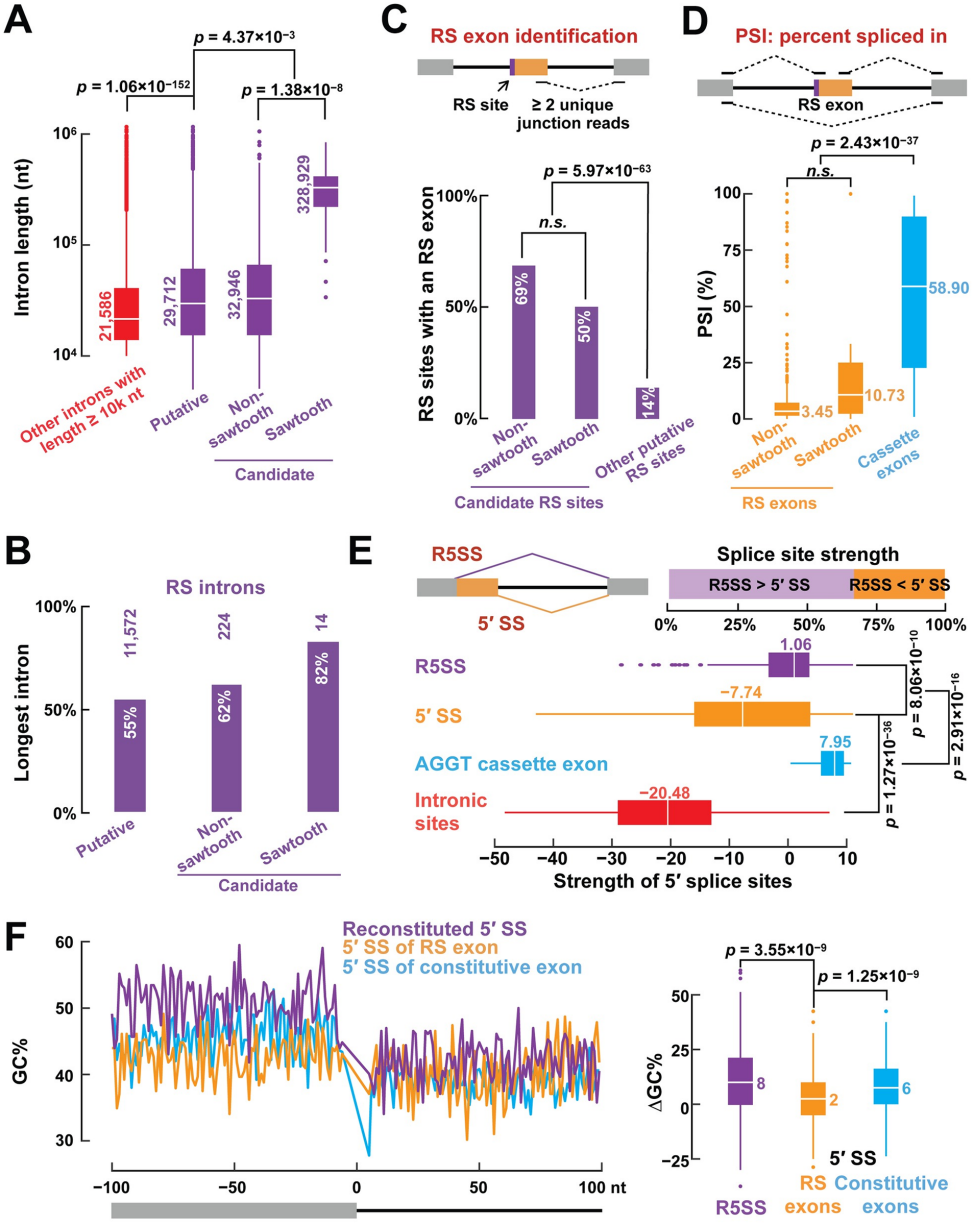
Sequence features of recursive splicing in humans. (A) RS sites tend to reside in long introns. Introns with putative, candidate or sawtooth RS sites have increasing lengths (Wilcoxon rank-sum tests). (B) Most introns with putative, candidate or sawtooth RS sites correspond to the longest introns of their genes. (C) We predict that more than 50% of candidate RS sites are followed by an RS exon supported by at least two unique 4sUDRB-seq junction reads, a significantly higher percentage than the putative RS sites that are not candidate RS sites (Fisher’s exact test). (D) The Percent-Spliced-In (PSI) values of RS exons are significantly lower than those of cassette exons according to steady-state RNA-seq data (Materials and methods; Wilcoxon rank-sum test). (E) Reconstituted 5′ splice sites are significantly stronger than the 5′ splice sites of RS exons (Wilcoxon signed-rank test)—67% of reconstituted 5′ splice sites are stronger than the 5′ splice sites of their corresponding RS exons. Both of these two groups of 5′ splice sites are significantly stronger than randomly chosen intronic sites but weaker than cassette exons with the AGGT motif (Wilcoxon rank-sum test). (F) Left: on average, the exons upstream of recursive 5′ splice sites (purple) have higher GC% than their downstream introns, like canonical exon-intron boundaries (blue), while there is no apparent GC% difference around the exon-intron boundaries of RS exons (orange). Right: reconstituted 5′ splice sites have significantly larger GC% difference than the 5′ splice sites of RS exons (medians are shown along with Wilcoxon signed-rank test *p*-values).

We first tested whether RS introns tended to be longer than non-RS introns. A direct comparison is not appropriate because we only searched for RS sites in long introns (≥ 5k nt). As specified in our pipeline (S2 Fig), candidate RS sites are those putative RS sites supported by RS junction reads and sawtooth RS sites are those candidate RS sites that show a sawtooth pattern, and neither of these additional requirements favors longer introns over shorter introns. Therefore, we compare the lengths of candidate RS introns with or without the sawtooth pattern with the lengths of putative introns. We found that candidate RS introns with the sawtooth pattern were significantly longer than the introns without the sawtooth pattern, which were in turn significantly longer than introns with putative RS sites (median lengths 328.9 k, 32.9 k, and 29.7 k; Wilcoxon rank-sum test *p*-values = 1.38×10^−8^ and 4.37×10^−3^ respectively; Fig 3A). Furthermore, 82% of the introns with sawtooth RS sites correspond to the longest introns of their genes, compared with 62% for candidate RS introns without the sawtooth pattern and 55% for putative RS introns (Fig 3B). Although most introns contain just one RS site, introns with two or more RS sites are significantly longer than introns with one RS site (S6A Fig, bottom). These results support the notion that long introns are enriched for RS sites, and recursive splicing is a mechanism aiding the removal of long introns.

RS exons were reported to be essential for the recognition of human RS sites according to the exon definition mechanism [4]; therefore, we next examined whether our predicted RS sites preceded an RS exon. Splice junction analysis (Fig 3C, top; Materials and methods) predicted that 69% of non-sawtooth and 50% of sawtooth RS sites were followed by an RS exon, which was significantly more frequent than those putative RS sites that were not candidate RS sites (14%; Fisher’s exact test *p*-value = 5.97×10^−63^; Fig 3C). Furthermore, the regions around the 5′ splice sites immediately downstream of the predicted RS exons are evolutionarily conserved, albeit not to the same extent as canonical exons (S6B Fig), lending support for these RS exons. We also detected multiple potential 5′ splice sites following RS exons, significantly more frequently than following random intronic sites (Wilcoxon rank-sum test *p*-value = 3.65×10^−2^; S6C Fig). These additional 5′ splice sites may provide further opportunities to facilitate the splicing of the RS exons.

Most previously identified RS exons are spliced out of the final transcripts, and a competition model was proposed to explain this result: the reconstituted 5′ splice site at an RS site outcompetes the 5′ splice site of the RS exon [4]. Indeed, the median Percent-Spliced-In (PSI) value [15] for our predicted non-sawtooth and sawtooth RS exons are only 3.45 and 10.73, respectively, much lower than that of cassette exons (58.90; Wilcoxon rank-sum test *p*-value = 2.43×10^−37^; Fig 3D). Accordingly, 67% of the reconstituted 5′ splice sites at the RS sites are stronger than the 5′ splice site of the corresponding RS exons (Wilcoxon signed-rank test *p*-value = 8.06×10^−10^; Fig 3E), supporting the competition model. Both of these two groups of 5′ splice sites are significantly weaker than the cassette exons that match the AGGT RS site motif, indicating that most cassette exons are not RS exons (Wilcoxon signed-rank test *p*-values = 2.91×10^−16^, respectively; Fig 3E).

To further investigate the low efficiency of RS exon inclusion, we analyzed the GC contents around RS exons. Short human exons tend to have higher GC% than their long flanking introns. The exon definition model suggests that this differential GC signal assists the spliceosome to recognize the short exon in the vast intronic landscape and accordingly, exons with greater GC differences from their flanking introns are included at higher rates despite the often weaker 5′ and 3′ splice sites surrounding these exons [16]. In sharp contrast to constitutive exons which have substantially higher GC% than their downstream introns, RS exons exhibit the same GC% as their downstream introns, while the exons upstream of RS exons show even more elevated GC% over their reconstituted introns than constitutive exons over their downstream introns (Fig 3F). This substantial difference in GC% between RS exons and their upstream exons (median 2% vs. 8%; Wilcoxon signed-rank *p*-value = 3.55×10^−9^) further favors the upstream exons in the competition and accounts for the inefficiency of RS exon inclusion.

Few minor isoforms, if any, can still be detected with RS exons (Fig 3C and 3D). We asked whether these isoforms would be translated into mature proteins or introduce premature termination codons (PTCs), which might result in their rapid degradation via nonsense-mediated decay (NMD) [4]. NMD depends on the exon-exon junction complex (EJC), and an efficient NMD requires a PTC to be located at least 50 nucleotides upstream of the 3′ most exon-exon junction (EEJ) [17, 18]. Indeed, 68% of RS exons would introduce a PTC more than 50 nucleotides upstream of the 3′ most EEJ (S6D Fig), suggesting that NMD is another factor that contributes to the low abundance of RS exons in mature transcripts.

### Many recursive splicing events occur post-transcriptionally with long delays but proceed in a timely fashion

Ample evidence supports the co-occurrence of and interaction between transcription and splicing [7, 19–21]. It is not known whether recursive splicing is kinetically coupled with Pol II elongation, although this is possible because recursive splicing is one of the splicing strategies; thus, we used the time-course 4sUDRB-seq data to test this possibility.

**Fig 4:**
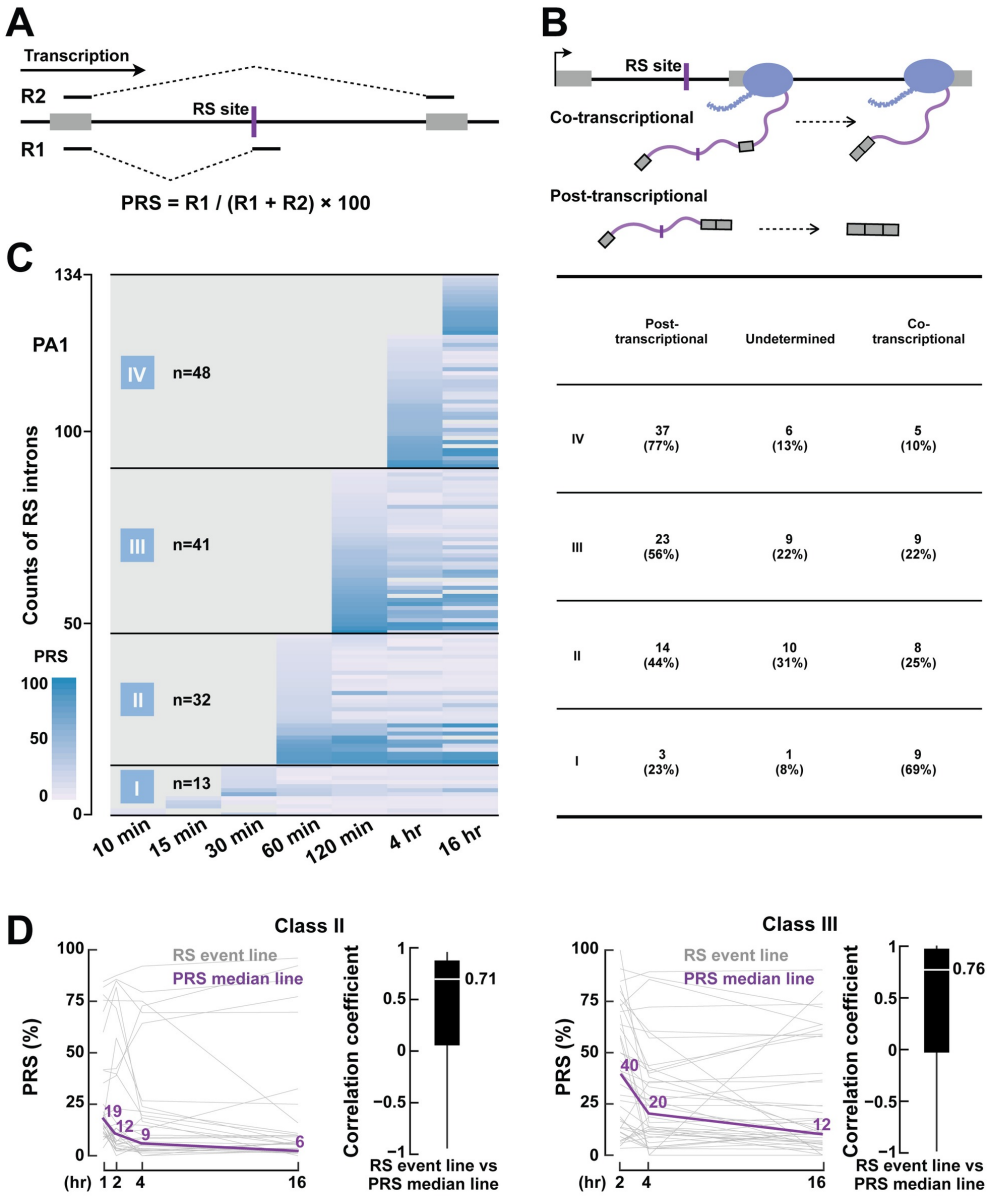
Recursive splicing occurs with a delay after transcription. (A) Percent Recursive Splicing (PRS) is defined as the percentage of junction reads that support the intermediate product of recursive splicing. (B) A schematic diagram to show the definition of co-transcriptional and post-transcriptional recursive splicing. For co-transcriptional recursive splicing, the recursive splicing finished before the entire gene was transcribed (top panel), while in post-transcriptional recursive splicing, recursive splicing started after the gene was transcribed (bottom panel). (C) The landscape of recursive splicing during transcription in PA1 cells. (Left) Each row is an RS event. All RS events are clustered into four classes according to their onset time for recursive splicing and sorted within each class by PRS. (Right) The table lists the counts and percentages of RS intron with post-transcriptional or co-transcriptional recursive splicing. For some RS events, we could not determine whether they were post- or co-transcriptional due to the lack of time resolution. (D) PRS dropped progressively after the onset of recursive splicing. Class II (left panel) and III (right panel) introns are shown and the median PRS time course is shown as purple lines. The boxplots show correlation coefficients between the PRS time course of individual RS events and the median time course.

Historically, co-transcriptional splicing is interpreted as splicing that takes place after an intron is transcribed but before the transcription of the gene is completed and the transcript is released, while post-transcriptional splicing takes place after transcription termination. Here, we follow this tradition and define co-transcriptional recursive splicing as the detection of a junction read that spans the recursive splice site (the R1 read in Fig 4A) before the gene is completely transcribed (Fig 4B). Because the 4sUDRB-seq data were sampled at specific time points, to determine whether an RS event was co-transcriptional, we first identified the earliest time point that the RS event could be detected and then compared this time point with the amount of time needed by Pol II to transcribe the entire gene. We used the 4sUDRB-seq data to estimate the transcription rate for each gene, and the averages were 2.52 kb/min for PA1 cells, 2.40 kb/min for FB cells, and 2.28 kb/min for H9 cells, in good agreement with previous estimates [22, 23].

To quantify an RS event, we further defined PRS (Percent Recursive Splicing; Materials and methods) as a function of the junction reads R1 and R2 to evaluate the percentage of transcripts that completed the first step of recursive splicing (Fig 4A). We clustered the 134 RS events in PA1 cells by their earliest time points of onset (indicated by a positive PRS): within 1 hour (class I), 1–2 hours (class II), 2–4 hours (class III), or after 4 hours (class IV; Fig 4C, left panel). For each class, we determined the percentage of RS events that commenced after the gene was completely transcribed (S7A Fig; Materials and methods). Most RS events (121 of 134 in PA1 cells) belonged to classes II to IV, i.e., they commenced at least one hour after Pol II elongation. Surprisingly, for many of these RS introns, the recursive splicing is post-transcriptional (Fig 4C, right panel). For example, for Class II RS events, the RS junction reads started to appear at 60 min, but the transcription of 44% of the corresponding genes was completed in less than 30 mins (Fig 4C, right), indicating that at least 44% of Class II events in PA1 cells were post-transcriptional. Similar results were also observed in Class III: 56% of the events in this class started recursive splicing after the gene was completely transcribed. We applied the same analysis to H9 and FB cells, which had a shorter time course of 4sU labeling than PA1 cells (2 hr versus 16 hr), and observed that at least one-third of RS events underwent post-transcriptional recursive splicing (S7B Fig, bottom).

The sawtooth pattern in RS introns is predicated on the assumption that recursive splicing rapidly follows transcription. However, if the intron is spliced with a long delay after transcription, the sawtooth pattern would be diminished. Thus, we asked whether we were more likely to detect the sawtooth pattern for co-transcriptional RS introns than for post-transcriptional RS introns. Among the 12 sawtooth RS introns in PA1 cells, we classify eight as co-transcriptional and none as post-transcriptional, and we did not have the time resolution to classify the remaining four. Overall, we could detect the sawtooth pattern for 26% of the 31 PA1 co-transcriptional introns, much higher than for the post-transcriptional introns (0%, 0 out of 77, Fisher’s exact test p-value = 2.24×10^−5^; S7C Fig). These results indicate that the sawtooth pattern is only observed at a subset of RS sites partly because recursive splicing is often a post-transcriptional event.

Given that the onset of recursive splicing lags behind the transcription of RS introns, we asked whether recursive splicing precedes slowly as well. To answer this question, we compared the PRS at each time point for the RS introns in Class II and III. We observed a sharp initial decrease of PRS followed by a slower decline, with the median PRS of Class II RS events halving by the end of the first hour and quartering after two more hours (Fig 4D). The decrease in RPS between the first two time points is significantly higher than the decrease between later time points (S7D Fig). In summary, these results suggested that although most RS events start after the completion of transcription with a considerable delay, they proceed in a timely fashion henceforth.

### Genes that undergo recursive splicing tend to have fast Pol II elongation rates

RS genes have significantly higher Pol II elongation rates than non-RS genes in all three cell lines (S8A Fig; S5 Table). It has been reported that long genes tend to be transcribed more rapidly [24] and RS introns are long (Fig 3A), so we asked whether RS genes have other features of genes with fast Pol II elongation rates.

Multiple histone modifications have been reported to correlate with Pol II elongation. For humans, genes with higher elongation rates show higher densities of H3K79me2 and H4K20me1 throughout their bodies than genes with lower elongating rates [24], while for mouse, H3K79me2 and H3K4me1 are enriched only in the proximal regions around the TSS of fast elongating genes [25]. We observed similar results for RS genes. Among the histone marks assayed in H9-derived neural progenitor cells, H3K79me2, H4K20me1 and H3K4me1 show higher signals at the first 20 kb of RS genes compared with non-RS genes (S8B Fig). We did not observe a higher signal of H3K36me3 in RS genes than non-RS genes (S8C Fig), consistent with previous studies which reported that gene-body H3K36me3 level was correlated with fast Pol II elongation in mice but not in humans [24].

We examined several genomic features known to be correlated with fast Pol II elongation in human [24] and mouse [25]—gene length and first intron length are positively correlated with fast Pol II, while exon density shows the most prominent negative correlation (S8D Fig). Compared with non-RS genes, RS genes in all three cell lines contain significantly longer first intron, lower exon density and more simple repeats (S8D Fig). Furthermore, RS genes have significantly lower GC content (S8D Fig), features shown to slow down Pol II elongation [24]. In summary, our results indicate that RS genes have high Pol II elongation rates and many genomic and epigenetic characteristics that are associated with fast elongation.

### Recursive splicing is not constitutive and is cell-type specific

It has been proposed that fly RS introns are constitutively spliced out via their RS sites, evidenced by the lack of junction reads that correspond to the lariats of the canonical splicing for these introns [2, 26]. However, it is not known whether recursive splicing is constitutive in humans and conflicting data have been reported: individually mutating three of seven RS sites in the first intron of human *SAMD4A* gene substantially reduced the splicing efficiency of this intron [26], while blockage of RS site usage in human *CADM1* and *ANK3* genes did not affect their expression levels [4]. We used the time-series 4sUDRB-seq data to test whether human RS introns were constitutively spliced by the recursive mechanism.

**Fig 5:**
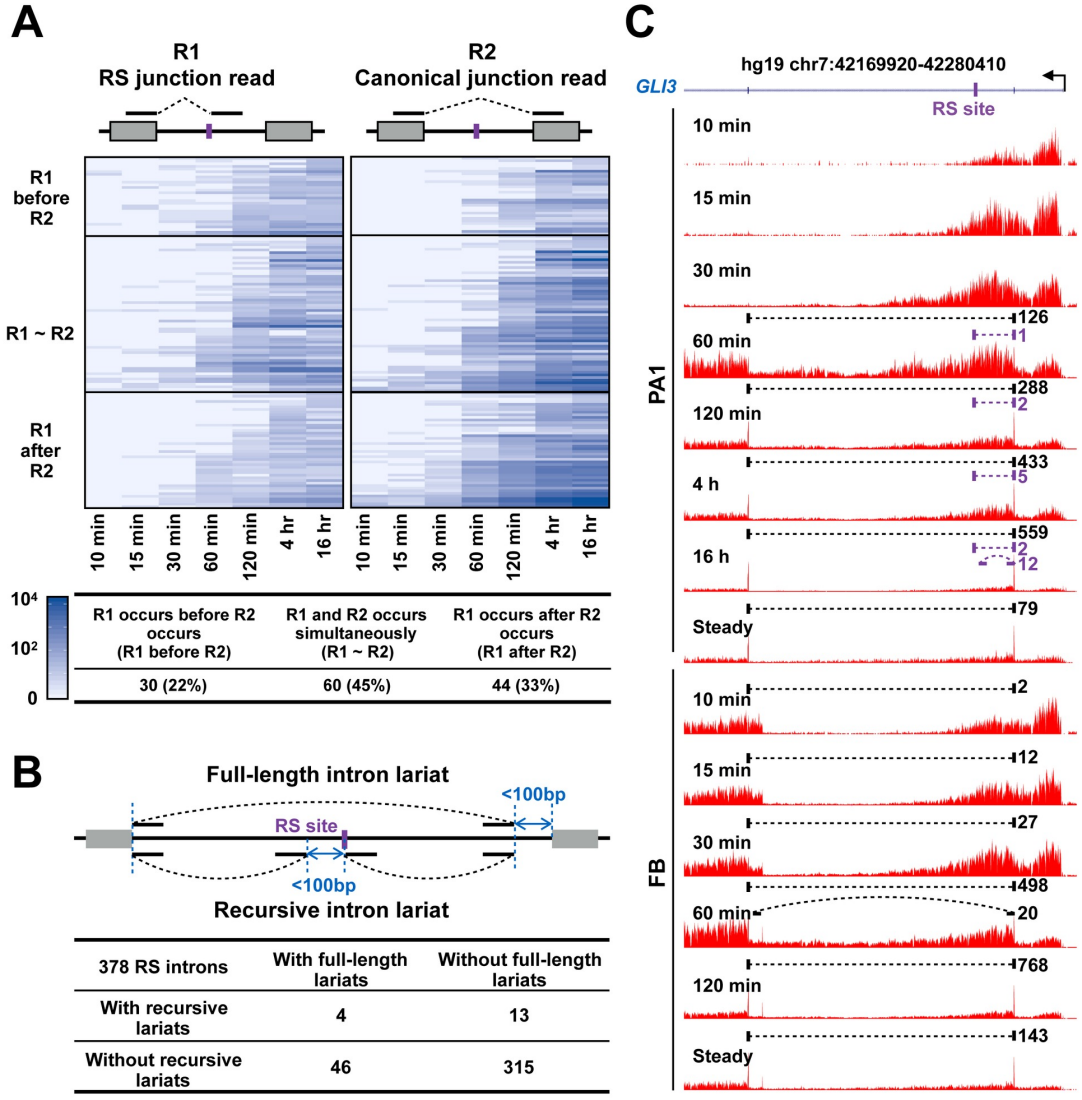
Recursive splicing is not constitutive and is cell-type specific. (A) Pairwise comparison of the occurrence of RS junction reads (R1) and canonical junction reads (R2) in RS introns. RS introns were classified into three group according to the appearance orders of R1 and R2. (B) Identification of full-length intron lariats (Materials and methods) in RS introns. (C) An example in the human *GLI3* gene shows that recursive splicing is cell-type specific. Junction reads of recursive splicing (purple) or canonical splicing (black) are depicted as dashed straight lines, and lariats reads are depicted as dashed curved lines.

We hypothesized that if recursive splicing were constitutive, we would detect junction reads corresponding to the first step of recursive splicing (R1, Fig 5A) before we detect junction reads corresponding to canonical splicing (R2). In PA1 cells, we detected R1 in an earlier time point than R2 for 22% of the RS events, R1 and R2 at the same time point for 45% of the RS events, and R1 after R2 for 33% of the RS events (Fig 5A). We obtained similar results for H9 and FB cells, detecting R1 at a later time point than R2 for 47% and 18% of the RS events respectively (S9A Fig). For RS events in which R1 and R2 appeared at the same time point, we detected more R2 reads than R1 reads (darker blue in the R2 heatmap than the R1 heatmap; Fig 5A and S9A Fig), suggesting that recursive and canonical splicing occur concurrently for these RS introns although we do not have the time resolution to conclude definitively.

We further searched for lariat reads that could support recursive splicing or canonical splicing of RS introns using publicly available RNA-seq data. Among the 378 RS introns, we could detect reads that traverse the predicted branch point in a lariat configuration (full-length or recursive introns; see Materials and methods) for 17% introns (63 out of 378). For 79% of these 63 introns with lariat-supporting reads, the lariats corresponded to canonical splicing, i.e., they spanned the entire length of the intron subsuming the RS site (Fig 5B), indicating that these introns are removed via the canonical splicing mechanism, i.e., recursive splicing is not constitutive for these introns. We further observed an example of cell-type-specific switching between recursive splicing and canonical splicing. The second intron of the *GLI3* gene was recursively spliced in PA1 cells, as supported by both junction reads for recursive splicing and lariat reads for recursive splicing at the 16-hr time point, whereas in FB neurons we could only find junction and lariat reads to support the full-length intron at the 60-min time point, suggesting that this intron is primarily spliced via the canonical mechanism in FB neurons (Fig 5C). Overall, we detected lariat reads that support the full-length intron in other cell lines for 34 PA1, 9 H9, and 15 FB RS introns in cells (S9B Fig). Collectively, these results suggested recursive splicing in humans, unlike in flies, is not mutually exclusive with canonical splicing and the choice between them may be under cell-type-specific regulation.

## Discussion

Recursive splicing is a non-canonical splicing process which removes an intron in multiple steps. It was first identified for a 73-kb intron of the fly *Ubx* gene [1] and recently shown to act on additional fly [2] and human introns [4]. In particular, Sibley et al. identified 435 putative human RS sites, including 9 RS sites that showed the sawtooth pattern in their steady-state RNA-seq data. We hypothesized that time-course 4sUDRB-seq data might be more effective than steady-state RNA-seq data for identifying RS sites and studying the temporal progression of recursive splicing. Thus, we built a computational pipeline to reanalyze the published 4sUDRB-seq data on three human cell lines [8]. We used the six RS sites by Sibley et al. that had sufficient expression in the 4sUDRB-seq data and did not overlap annotated cassette exons to set strict cutoffs in our pipeline. Analyzing only long introns (≥ 5k nt), we identified 342 candidate RS sites and 16 sawtooth RS sites. These include all 20 of the 435 putative RS sites by Sibley et al. that passed the stringent filtering steps of our pipeline. Thus, our pipeline substantially increased the number of candidate RS sites in long introns. If these criteria were relaxed, we would have predicted even more RS sites in humans. We performed detailed comparison and concluded that indeed 4sUDRB-seq data are more effective than steady-state RNA-seq data in finding RS sites.

We only searched for highly conserved RS sites in long introns that did not overlap cassette exons. If we had not excluded RS sites that overlapped cassette exons, we would have had 3,140 more putative RS sites. Some cassette exons were reported to also function as RS exons [4], but we found that cassette exons tended to have significantly stronger 5′ splice sites and much higher PSI values than RS exons (Fig 3D and 3E). Thus, we conclude that most cassette exons do not also function as RS exons.

In both human and fly, RS exons are crucial to the recognition of RS site [3, 4]. RS sites followed by an RS exon are subsequently spliced out via a competition mechanism[3, 4]. We found several lines of evidence that supported human RS exons and the competition between 5′ splice sites for skipping these RS exons. One feature that we report here for the first time is that the GC% of RS exons are typically as low as the GC% of their downstream introns while their upstream exons are even more GC-rich than canonical exons. Thus, the enlarged GC% difference between an RS exon and its upstream exon promotes the skipping of the RS exon.

Even with time-series 4sUDRB-seq data, we observed the sawtooth pattern for only 16 out of our 342 candidate RS sites, although the sawtooth pattern was consistently observed at multiple time points. This observation prompted us to examine the hypothesis underlying the sawtooth pattern, i.e., recursive splicing rapidly follows transcription. We found that for 57% of the PA1 RS introns, we could detect their junction reads that supported the first step of the recursive splicing only after the entire gene was transcribed, suggesting that these RS introns were recursively spliced post-transcriptionally. This long delay partly explains why we could detect the sawtooth pattern for only a small fraction of the RS introns. Accordingly, among the 12 sawtooth RS introns with the sawtooth pattern in PA1 cells, eight are co-transcriptional, and the other four undetermined due to the lack of time resolution. Thus, unlike canonical splicing, recursive splicing often occurs post-transcriptionally in humans.

Another difference between the fly and humans with regards to recursive splicing is that it has been suggested that recursive splicing is constitutive in the fly [2], but it is not known whether it is so in humans. Again, we searched for RS junction reads that could support recursive or canonical splicing of RS introns in the 4sUDRB-seq data. We also searched for lariat reads that could support recursive or canonical splicing in RNase-R-treated RNA-seq data. Notwithstanding the limited time resolution of the 4sUDRB-seq data, we could detect canonical splicing before recursive splicing for 18–47% of RS introns in the three human cell lines, indicating human RS introns are spliced by both recursive and canonical splicing mechanisms.

Our results raised the question of whether recursive splicing and canonical splicing of long human RS introns are co-regulated. We also showed that genes with RS introns had high Pol II elongation rates. It is tempting to speculate that when canonical splicing cannot catch up with the rapid transcription, recursive splicing is employed as a supplementary mechanism for removing these introns.

In summary, we used time-series 4sUDRB-seq data to expand the collection of long RS introns in humans. We also deduced that many of these RS introns are spliced post-transcriptionally and by both canonical and recursive splicing mechanisms. Our results reveal a complex landscape of RNA splicing and its regulation in the human transcriptome.

## Materials and methods

### Datasets and mapping the reads to the genome

We downloaded the 4sUDRB-seq (nascent transcripts) and steady-state ribo− RNA-seq (total RNA after ribosomal RNA removal) datasets derived from PA1, H9 and H9-differentiated forebrain (FB) neuron progenitor cells from the Gene Expression Omnibus (GEO) with the accession GSE73325 [8]. To generate these 4sUDRB-seq data, Zhang et al. first incubated the cells with DRB for three hours to block Pol II elongation, and then labeled the newly transcribed RNAs with 4sU upon DRB release. At each time point after the synchronized start of transcription elongation, Zhang et al. extracted 4sU-labeled RNAs to perform RNA sequencing. Total RNA (100–140 μg) was used for biotinylation and purification of 4sU-labeled nascent RNAs. Corresponding 4sUDRB-seq samples with RNase R treatment were also downloaded from the same GEO entry to facilitate intron lariat analysis. The 4sUDRB-seq datasets of each cell line contain five time points of 4sU exposure (10, 15, 30, 60, and 120 min). Also, the PA1 4sUDRB-seq datasets include two more 4sU exposure durations (4 and 16 hr). As previously reported [8], these datasets have a low bias for the collection of nascent RNAs and high efficiency in measuring transcription elongation rates. We mapped the sequence reads from each sample using the HISAT2 tool [27] (Version 2.0.4) against the GRCh37/hg19 human reference genome and annotated the mapped reads with the UCSC Known Genes annotation (hg19 knownGene.txt updated on June 30, 2013).

To compare the performance of 4sUDRB-seq and ribo− RNA-seq data in identifying recursive splicing (RS) sites without being affected by sequencing depth, we randomly selected the same number of reads for each dataset and ran our RS site identification pipeline and downstream analysis with the same parameters (S3B and S5 Figs).

### A pipeline for identifying recursive splice sites

Previous studies revealed that RS sites have four genomic features [2, 4]. First, they tend to be in long introns. Second, they contain a sequence motif (AGGT), which is like the concatenated 3′ and 5′ splice site consensus motifs. Third, they are recognized by the spliceosome as a 3′ splice site in the first step of recursive splicing, so RS sites typically score well as 3′ splice sites. Fourth, known human and fly RS sites have high evolutionary conservation. To accurately identify RS sites genome-wide, we first compared the nine known human RS sites [4] and canonical splice sites on these four genomic features to determine the suitable cutoffs for our pipeline. Half of the 22,144 human annotated genes (hg19 knownGene.txt updated on June 30, 2013) have their median-length introns more than 1,928 nt long (S1A Fig, left) and their longest introns more than 10,090 nt long (S1A Fig, right). Thus, we chose 5 kb as the cutoff for defining long introns to focus our analyses on. Splicing strength analysis revealed that all the nine reported human RS sites have strong (≥ 5) splicing strength when evaluated as 3′ splice sites, which are even stronger than constitutive exons and cassette exons (S1B Fig). Also, these nine human RS sites are as conserved as canonical splice sites (conservation score ≥ 0.5) (S1C Fig). Taking into account these pieces of information, our pipeline contains three steps (S2 Fig), described as follows.

Step 1: We first identified all the sites with the RS site motif (AGGT) that were located in long introns (≥ 5k nt long according to UCSC Known Genes annotation) and more than 2k away from any annotated splice sites. Among the identified sites, those with low splicing strength (< 5) when judged as potential 3′ splice sites were filtered out. Furthermore, only the sites with high evolutionary conservation were retained (PhastCons score ≥ 0.5 computed for the AGGT four nucleotides using hg19.100way.phastCons.bw updated on Feb. 9, 2014). Finally, the sites that overlapped with any annotated splice sites or cassette exons were excluded. The sites that passed Step 1 are called *putative RS sites*.
Step 2: We used a mapping strategy similar to a previous work [2], which were configured for identifying RS junction reads. We first constructed a set of potential RS junctions by concatenating the last 95 nucleotides of the upstream exon with the 95 nucleotides downstream of each putative RS site. Sequence reads in these datasets are single ended and 100-bp long, so the reads that map to a junction would need to have an overhang of at least 5 nucleotides, ensuring that they are junction reads. We first aligned all reads directly against the GRCh37/hg19 human reference genome and discarded the reads that mapped. We then mapped the remaining reads to the junction database using Bowtie (Version 1.1.2) with the following parameters: −v 2 −k 5 −M 5 −−best. RS sites that had at least three unique junction reads with distinct offsets were then promoted to *candidate RS sites*.
Step 3: Known RS sites show a sawtooth pattern in RNA-seq data, characterized as a sharp increase of read density at the beginning of each intron and then a gradual decrease of read density in the direction of transcription until the next intron (S1D Fig). The sawtooth pattern was first observed for canonical splicing and attributed to co-transcriptional splicing [13]. To determine whether there is a sawtooth pattern at a candidate RS site, we first split the region surrounding each RS site (± 10k nt) into 1,000-nt-long bins and computed the average read density for each bin. We then identified sawtooth RS sites according to the following criteria. First, there must be at least a 2-fold difference in read density between the upstream and downstream regions—an increase for the genes on the plus strand and a decrease for the genes on the minus strand. Second, a permutation test was used to evaluate the consistency of read density difference before and after the candidate RS site, and a *p*-value less than 0.01 was deemed statistically significant. Finally, all the identified sawtooth patterns were manually inspected to remove false positives. Note that the RS sites that are spliced post-transcriptionally may not show a prominent sawtooth pattern; hence, this step is optional, and the sites that pass this step are called *sawtooth RS sites*.

### Characterization of RS sites

We compared introns harboring RS sites (called RS introns) with other introns (≥ 10k nt) that do not harbor RS sites (non-RS introns) in terms of length (Fig 3A).

We predicted RS exons based on junction reads detected in the 4sUDRB-seq datasets with the following steps. First, we used 4sUDRB-seq reads to identify all possible exon-exon junctions that could result in an RS exon up to 500-nt long (the 500-nt parameter was chosen based on the knowledge that 87% annotated human exons are shorter than 500 nt). We further required that there were at least two unique reads that mapped to the junction. If there were more than one putative 5′ splice site, the 5′ splice site with the most junction reads was defined as the 5′ splice site of the RS exon (Fig 3C). We estimated the Percent-Spliced-In (PSI) value [15] for each predicted RS exon, i.e., the frequency that the RS exon could be included in mature transcripts, using the steady-state RNA-seq data (Fig 3D).

When we computed the differential GC content between neighboring exons and introns (Fig 3F), we excluded the ±5 bp window around each splice site from the calculation, because we did not want to be biased by the splice site motifs.

### Identification of intron lariats

We identified reads that map to intron lariats and pass over the branch point using a strategy similar to previously described [28]. Briefly, we first mapped all RNA-seq reads to the human genome (hg19) using STAR [29] and only retained the unmapped reads. We then built an index using the first 23 nt sequence of each annotated intron (GENCODE release 19 supplemented with the introns flanking our RS sites) and mapped the unmapped reads to these 23-nt intron prefixes using Bowtie2 [30]. The portion of a read that maps to an intron prefix is defined as the A portion, and its upstream sequence is defined as the B portion. In the case of a lariat read, the B portion should map to the sequence upstream of the branch point of the intron that A portion belongs to. We required the B portion to be > 23 nt and mapped them to the human genome using Bowtie2, and only kept alignments with mapping qualities (MAPQ) ≥ 10. We then required that the A and B portions of the same read to be aligned to the same intron. The 3′ end of each B portion indicated the putative branch point. To filter out spurious alignments that might have risen from sequence errors or repetitive regions of the genome, we then reconstructed 200-nt putative lariat-template sequences (100 nt upstream of each putative branch point and the first 100 nt of its intron). We mapped these putative lariat-template sequences to the human genome and only retained those with ≤ 80% sequence identity for further analyses. To obtain the final annotation of branch points and their supporting reads, we aligned all reads (unmappable to the genome) to such lariat-template sequences and retained alignments that had at most one mismatch and ≥ 23 nt overhangs flanking the branch point. Finally, we only kept those branch points that were near (<100 nt) 3′ splice sites or RS sites (S4C Fig; Fig 5B).

### Splicing motif analysis

Splice site strength was calculated using MaxEntScan [11]. To estimate the splicing strength of reconstituted 5′ splice site upon recursive splicing (Figs 2B and 3E), we concatenated the last three nucleotides of the upstream exon with the six nucleotides downstream a candidate RS site and used the resulting sequence for the calculation. RS site motifs (S4D Fig) were created using WebLogo3 [31]. Branch points (Fig 2A) were predicted using SVM-BPfinder [32]. Consensus 5′ splice sites (S6C Fig) were the sites that matched GTAAG, GTGAG, GTAGG, GTATG, GTAAA, GTAAT, GTGGG, GTAAC, GTCAG, or GTACG.

### Gene Ontology enrichment analysis

Gene Ontology (GO) analysis was performed using the DAVID tool (https://david.ncifcrf.gov/, [33]), and GO terms from GOTERM_BP_FAT and GOTERM_MF_FAT were used. GO terms with multiple-testing-corrected *p-value*s [34] lower than 0.05 were deemed enriched (S3C Fig).

### Cell culture, RT-PCR, and Sanger sequencing for a predicted RS site (Fig 2C)

A549 cells were cultured as previously described [35]. Total RNA was purified using the RNeasy Mini Kit (Qiagen). cDNA was synthesized using SuperScript (ABI) and used as the template in RT-PCR. LA-Taq (Clontech) PCR kit was used for RT-PCR. PCR bands were gel purified (Qiagen), TOPO cloned (Invitrogen TOPO kit), and sent for sequencing (Genewiz). PCR primers are listed in S6 Table.

### Evaluation of the temporal progression of recursive splicing

To facilitate quantitative analysis of the recursive splicing process during Pol II transcription elongation, we define the PRS metric (Percent Recursive Splicing; Fig 4A) as the ratio between the number of RS junction reads (R1) and the total junction reads (R1 + R2). Defined as such, PRS quantifies how complete the first step of recursive splicing is. PRS first increases, indicating that the first step of recursive splicing starts to occur, and then it decreases, indicating that recursive splicing transitions from the first step to the second step. To simplify our analysis of the temporal progression of recursive splicing and decrease false positives (Fig 4 and S7 Fig), we only included the RS introns that had only one RS site and further met the following criteria:

1. R1 + R2 ≥ 10
2. 10 < PRS < 90 for at least one time point
3. When the PRS of an RS intron was computed for a particular time point, Pol II needed to have passed the RS site at this time point according to the estimated transcription elongation rate of this gene.

We estimated the Pol ll transcription elongation rate (TER) for each gene in each sample using the TERate tool [8] (https://github.com/YangLab/TERate). We estimated the rates from the two datasets with 10 or 15 min of 4sU labeling after DRB removal. For those genes without an estimated elongation rate at both time points due to being too short or having too few reads, an average Pol II elongation rate of 2,520 nt/min was used.

To determine whether a recursive splicing event occurred co-transcriptionally or post-transcriptionally, we compared the first time point of detecting a junction read that spanned the RS site with the time required for the complete transcription of the entire gene, using the estimated elongation rate for the gene (Fig 4B). Note that we can only score the final ligation product of recursive splicing and this ligation step may be delayed relative to the determination of splice site pairs, we may underestimate the number of co-transcriptional splicing. Due to the limited time resolution of the 4sUDRB-seq experiments (sampled at 10, 15, 30, 60, 120, 240, 960 mins), we could not classify all RS events into the co- or post-transcriptional classes. For example, if the gene is completely transcribed in 80 mins and we detected the onset of recursive splicing (indicated by a positive PRS) in the 120-min sample (i.e., not in any of the earlier samples), this recursive splicing event could have occurred before or after the entire gene was transcribed at 80 mins (we only know that it was after 60 mins and before 120 mins). To classify RS events into co- and post-transcriptional categories unambiguously, we tabulated the precise relationship between the onset time point of a positive PRS and the time point when the entire gene was transcribed (S7A Fig).

### Comparison of genomic and epigenetic features

Genomic features, including exon density, length of the first intron, the fraction of simple repeats, and GC content (S8D Fig), were calculated based on the UCSC Known Genes annotation (hg19 knownGene.txt updated on 2013/6/30) and RepeatMasker annotation (hg19 rmsk.txt.gz updated on 2009/4/27). The ChIP-seq data of several histone modifications on neural progenitor cells derived from the H9 embryonic stem cells were downloaded from the ENCODE Portal (https://www.encodeproject.org/; accessions for H3K79me2: ENCFF905UHS, H4K20me1: ENCFF560PIQ, H3K4me1: ENCFF637CXU, and H3K36me3: ENCFF555VCB). We plotted normalized ChIP-seq signals, i.e., fold change over control, for RS introns in S8B and S8C Fig.

### Data access

The source code for our RS site identification pipeline and downstream analyses can be accessed from the GitHub (https://github.com/kepbod/rs).

## Supporting information

S1 Fig Genomic features of all human introns and previously reported RS introns. (A) Length distribution of human introns. (Left) The cumulative distributions of human genes by the shortest, median-length, or longest intron of each gene. (Right) A breakdown of human genes by their longest introns. (B) The splicing strengths of the 3′ splice sites of the nine reported human RS sites are higher than 5 (labeled with a dashed line), comparable with the 3′ splice site strength of cassette exons and constitutive exons. (C) The evolutionary conservation (PhastCon) scores of the nine reported human RS sites are higher than 0.5 (labeled with a dashed line), comparable with the conservation of cassette exons and constitutive exons. (D) The nine reported RS sites show large read density differences in the forebrain steady-state ribo− RNA-seq dataset. A ± 10 kb region centered on each RS site is examined for read density (normalized as a Z score and displayed as a heat map with each column being a 1 kb bin). Note that although all nine RS sites show obvious sawtooth pattern in the RNA-seq dataset, only six of them (red) were predicted by our pipeline, because we did not detect sufficient RS junction reads for two sites (Materials and methods), and the third site overlaps an annotated cassette exon, so it was filtered out by our pipeline (in black).
(PDF)

S2 Fig A computational pipeline for systematically identifying RS sites. Potential RS sites were first identified according to six genomic features (Step 1), and RS junction reads were then identified using a custom-built junction index (Step 2), resulting in candidate RS sites. For each candidate RS site, the presence of a sawtooth pattern was evaluated with a series of criteria, and sawtooth RS sites with an obvious sawtooth pattern were selected.
(PDF)

S3 Fig Genome-wide identification of human RS sites using 4sUDRB-seq data. (A) Sequencing depths of 4sUDRB-seq datasets used in this study. (B) More RS sites were identified in FB neurons than H9 cells using the 30 or 120 min 4sUDRB-seq samples at equalized sequencing depth. (C) Genes with recursive splicing are enriched in several gene ontology terms, in particular, neuron projection guidance and axon guidance.
(PDF)

S4 Fig Validation of identified RS sites. (A) Comparison between RS sites identified by previous study [4] and this study. We note that 12 of the 20 filtered RS sites (top panel) could be detected by our pipeline (bottom panel). (B) Sixty percent of putative RS sites and 89% of candidate RS sites could be detected with recursive splicing junctions using ENCODE total RNA-seq samples. (C) Identification of recursive splicing intron lariats to support the occurrence of recursive splicing (top panel, Materials and methods). The branch point identified by lariat reads showed proper distance to 3′ splice site (middle panel) and canonical sequence motif (bottom panel). (D) The sequence motifs of reconstituted 5′ splice site of candidate (top panel) and sawtooth (bottom panel) RS sites.
(PDF)

S5 Fig 4sUDRB-seq data are more effective than RNA-seq data for RS site identification. (A) At the same sequencing depth (80 M), more RS sites were identified in the 120 min 4sUDRB-seq datasets (red bars) than the steady-state RNA-seq datasets (blue bars) for PA1 and H9. (B) More RS sites and RS junctions were detected using the 4sUDRB-seq datasets with long 4sU labeling time (red bars) than the steady-state RNA-seq dataset (blue bars) in PA1 cells. (C) Most RS sites identified using the steady-state RNA-seq dataset were also found using 4sUDRB-seq (left Venn diagrams). The RS junctions were supported by comparable numbers of 4sUDRB-seq reads and RNA-seq reads at the same sequencing depth (right bar plots; outliers omitted for clarity). (D) The 4sUDRB-seq signal profiles at the *PDE4D* gene. The left panel shows three RS sites identified in PA1 cells, including two sites reported by Sibley et al. (RS1 and RS3, blue) and one novel site (RS2, purple). The right panel shows the numbers of RS junction reads supporting the three RS sites in the PA1 4sUDRB-seq data.
(PDF)

S6 Fig Genomic features of identified RS sites. (A) Most RS introns have one RS site (top panel), whereas introns with more than two RS sites are significantly longer than introns with one RS site (bottom panel; medians and Wilcoxon rank-sum test *p*-values are labeled). (B) The genomic regions around the 5′ splice sites of RS exons (orange) are more evolutionarily conserved than randomly chosen intronic regions (red), albeit not as conserved as constitutive exons (light blue) and cassette exons with the AGGT motif (dark blue). (C) RS sites are followed by more predicted 5′ splice sites than randomly chosen intronic sites are (Wilcoxon rank-sum test). (D) Sixty-eight percent of RS exons contain at least one premature termination codon located more than 50 nucleotides upstream of the last exon-exon junction.
(PDF)

S7 Fig The time course of recursive splicing evaluated against transcriptional elongation. (A) The criteria for defining post- and co-transcriptional recursive splicing. (B) The time courses of recursive splicing in H9 cells and FB neurons. (C) Among candidate RS sites, a higher percentage of co-transcriptional sites shows the sawtooth pattern than post-transcriptional sites (26% vs. 0%, *p*-value = 2.24×10^−5^, Fisher’s exact test). (D) PRS drops rapidly after the onset of recursive splicing (Wilcoxon rank-sum test).
(PDF)

S8 Fig Genes that undergo recursive splicing tend to have fast Pol II elongation rates. (A) The Pol II elongation rates of genes with RS introns (RS genes) are higher than those of non-RS genes with or without long introns in FB neurons (left panel), PA1 cells (middle panel), and H9 (right panel) cells. Medians and Wilcoxon rank-sum test *p*-values are labeled. (B) RS genes are enriched in several histone marks. Compared with non-RS genes (black lines), RS genes (purple line) have a higher density of histone modification (left panel: H3K79me2, middle panel: H4K20me1, right panel: H3K4me1) near the transcription start site (TSS). (C) There is no significant difference in H3K36me3 levels between RS genes (purple line) and non-RS genes (black line). (D) RS genes in FB neurons, PA1, and H9 cells show a series of genomic features that are correlated with fast Pol II elongation.
(PDF)

S9 Fig Recursive splicing is not constitutive. (A) A comparison of the occurrence of RS junction reads (R1) and canonical junction reads (R2) in H9 cells (left) and FB neurons (right). (B) Number of reads that support RS lariats or full-length lariats in RS introns.
(PDF)

S1 Table A list of putative RS sites. We identified 36,870 putative RS sites located in 21,202 introns in the human genome, and they are listed with gene symbol, intron coordinates, site locus, and related genomic features.
(XLSX)

S2 Table A list of candidate RS sites. In total, we identified 342 candidate RS sites in PA1, H9, or FB cells, and they are listed with gene symbol, intron coordinates, site locus, count of junction reads, and related genomic features.
(XLSX)

S3 Table A list of RS sites with sawtooth pattern. A list of RS sites with the sawtooth pattern in PA1, H9, or FB cells detected by a computer program. RS sites identified by Sibley et al. are colored red. Sixteen sawtooth RS sites confirmed by manual inspection are colored purple.
(XLSX)

S4 Table A list of lariat reads supporting recursive splicing. The accessions of ENCODE and GEO datasets are provided.
(XLSX)

S5 Table Transcription elongation rate of expressed gene estimated by 4sUDRB-seq in PA1, H9, or FB cells. We used 4sUDRB-seq data at two time points (10 min and 15 min) for the estimation and averaged the estimated rates. NA indicates that a rate could not be estimated because the gene is too short or has too few reads.
(XLSX)

S6 Table Primer sequences used for validating the RS site in Fig 2C. (XLSX)
