# Supplementary figures and images for: The temporal landscape of recursive splicing during Pol II transcription elongation in human cells
Source: PLoS Genet. 2018 Aug 27;14(8):e1007579. doi: 10.1371/journal.pgen.1007579 (PMC6110456; doi:10.1371/journal.pgen.1007579)

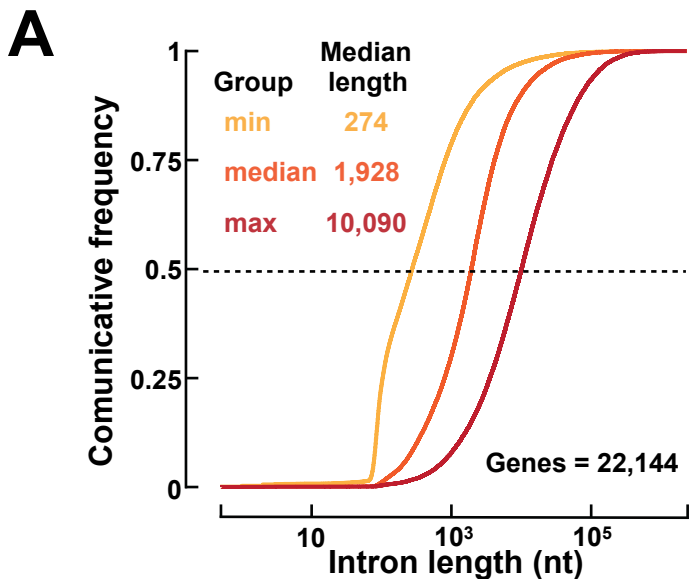

| Maximum intron length in a gene |                 |            |
|---------------------------------|-----------------|------------|
| Length (nt)                     | Number of genes | Percentage |
| < 10k                           | 11,021          | 50%        |
| 10k ~ 30k                       | 5,900           | 27%        |
| 30k ~ 50k                       | 1,938           | 9%         |
| 50k ~ 100k                      | 1,846           | 8%         |
| 100k ~ 150k                     | 643             | 3%         |
| ≥150k                           | 796             | 3%         |

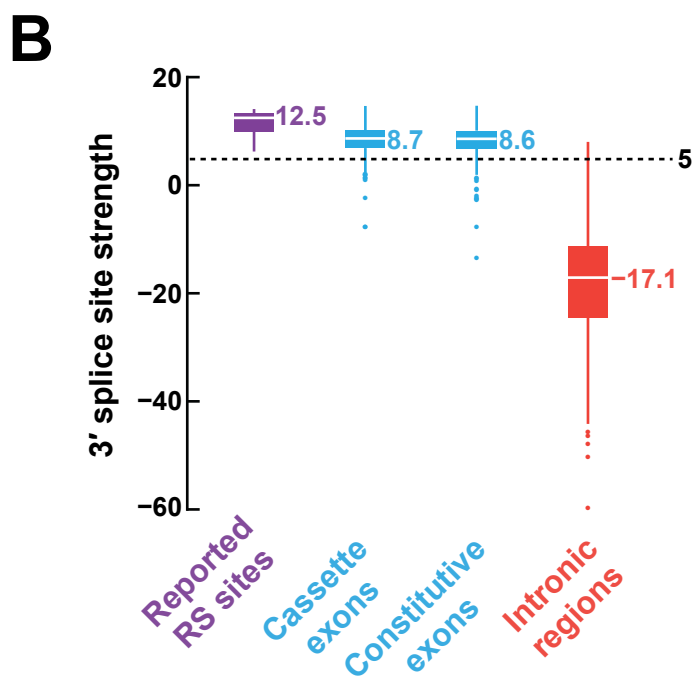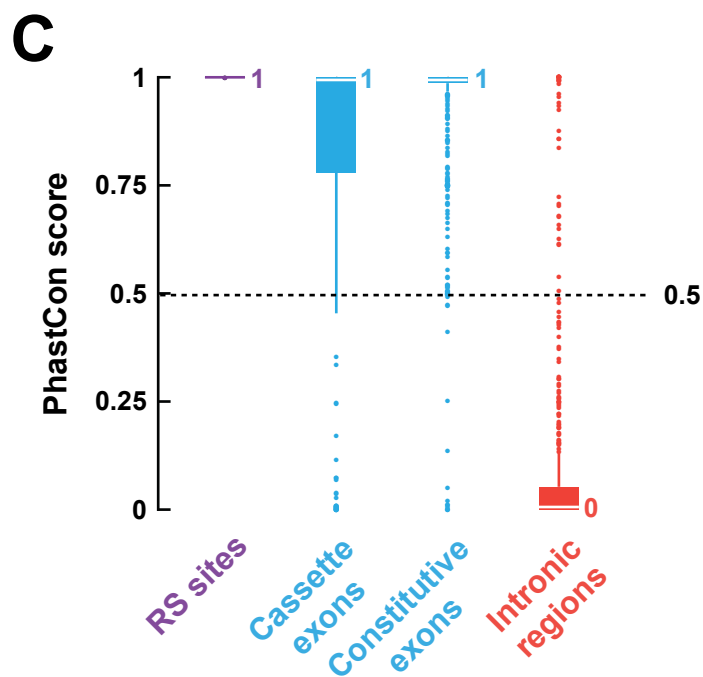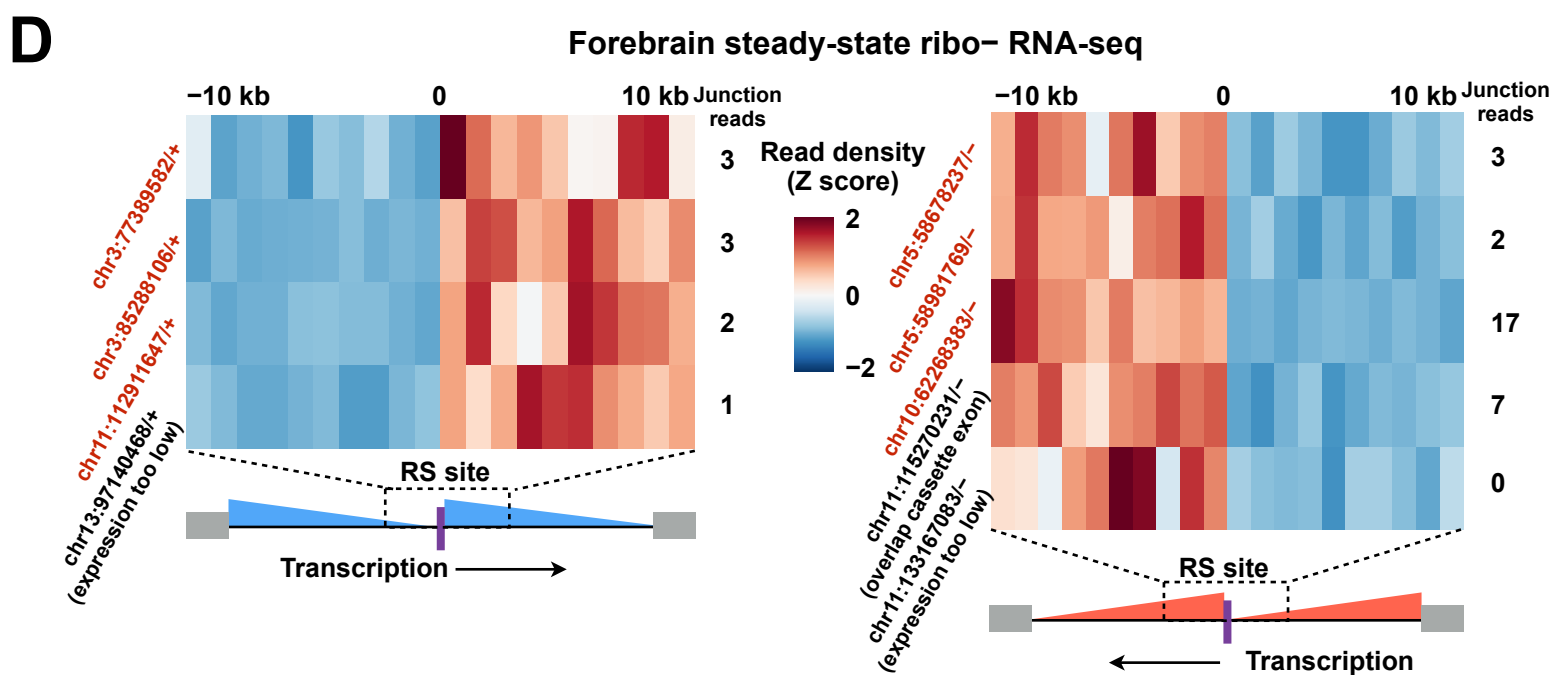

Supplement: S1 Fig — (A) Length distribution of human introns. (Left) The cumulative distributions of human genes by the shortest, median-length, or longest intron of each gene. (Right) A breakdown of human genes by their longest introns. (B) The splicing strengths of the 3′ splice sites of the nine reported human RS sites are higher than 5 (labeled with a dashed line), comparable with the 3′ splice site strength of cassette exons and constitutive exons. (C) The evolutionary conservation (PhastCon) scores of the nine reported human RS sites are higher than 0.5 (labeled with a dashed line), comparable with the conservation of cassette exons and constitutive exons. (D) The nine reported RS sites show large read density differences in the forebrain steady-state ribo− RNA-seq dataset. A ± 10 kb region centered on each RS site is examined for read density (normalized as a Z score and displayed as a heat map with each column being a 1 kb bin). Note that although all nine RS sites show obvious sawtooth pattern in the RNA-seq dataset, only six of them (red) were predicted by our pipeline, because we did not detect sufficient RS junction reads for two sites (Materials and methods), and the third site overlaps an annotated cassette exon, so it was filtered out by our pipeline (in black). (PDF) [file pgen.1007579.s001.pdf]

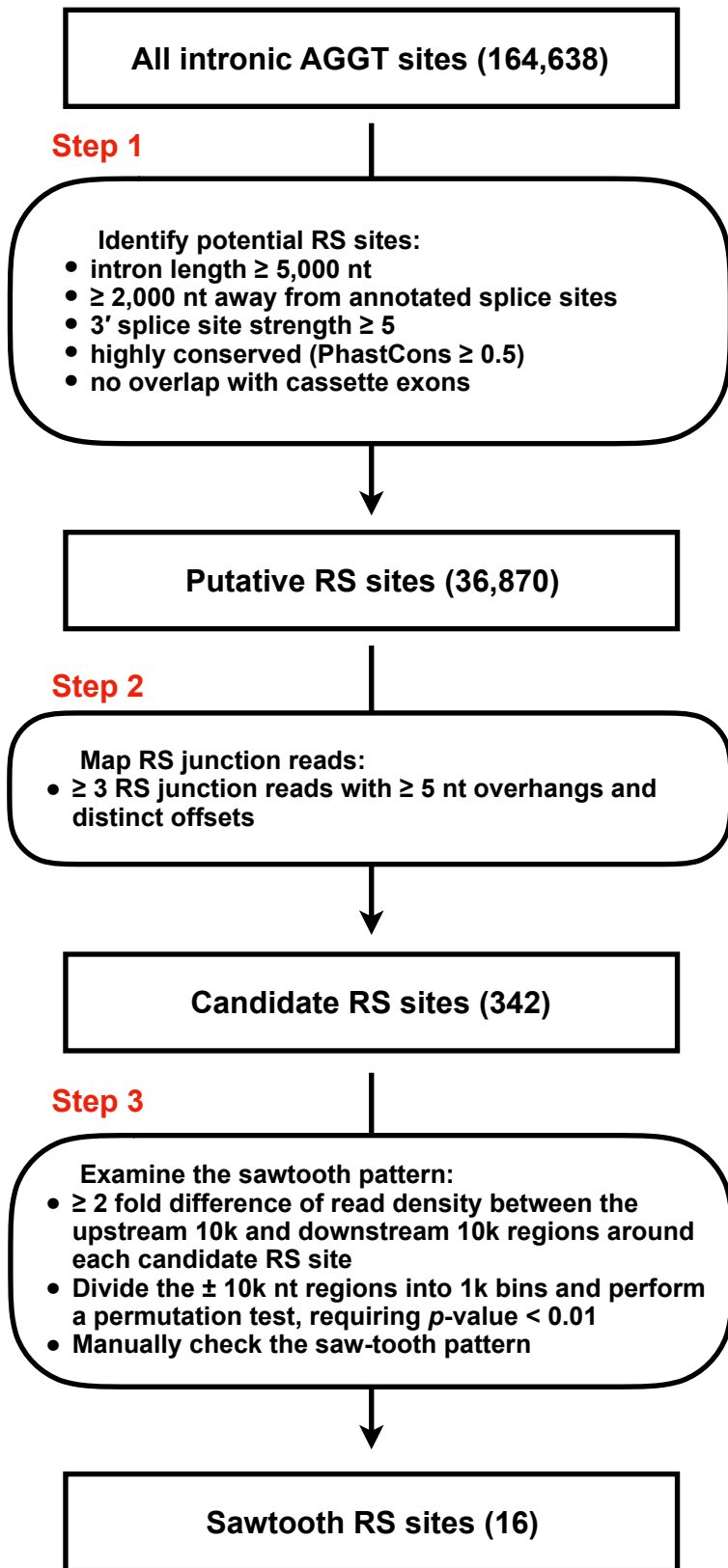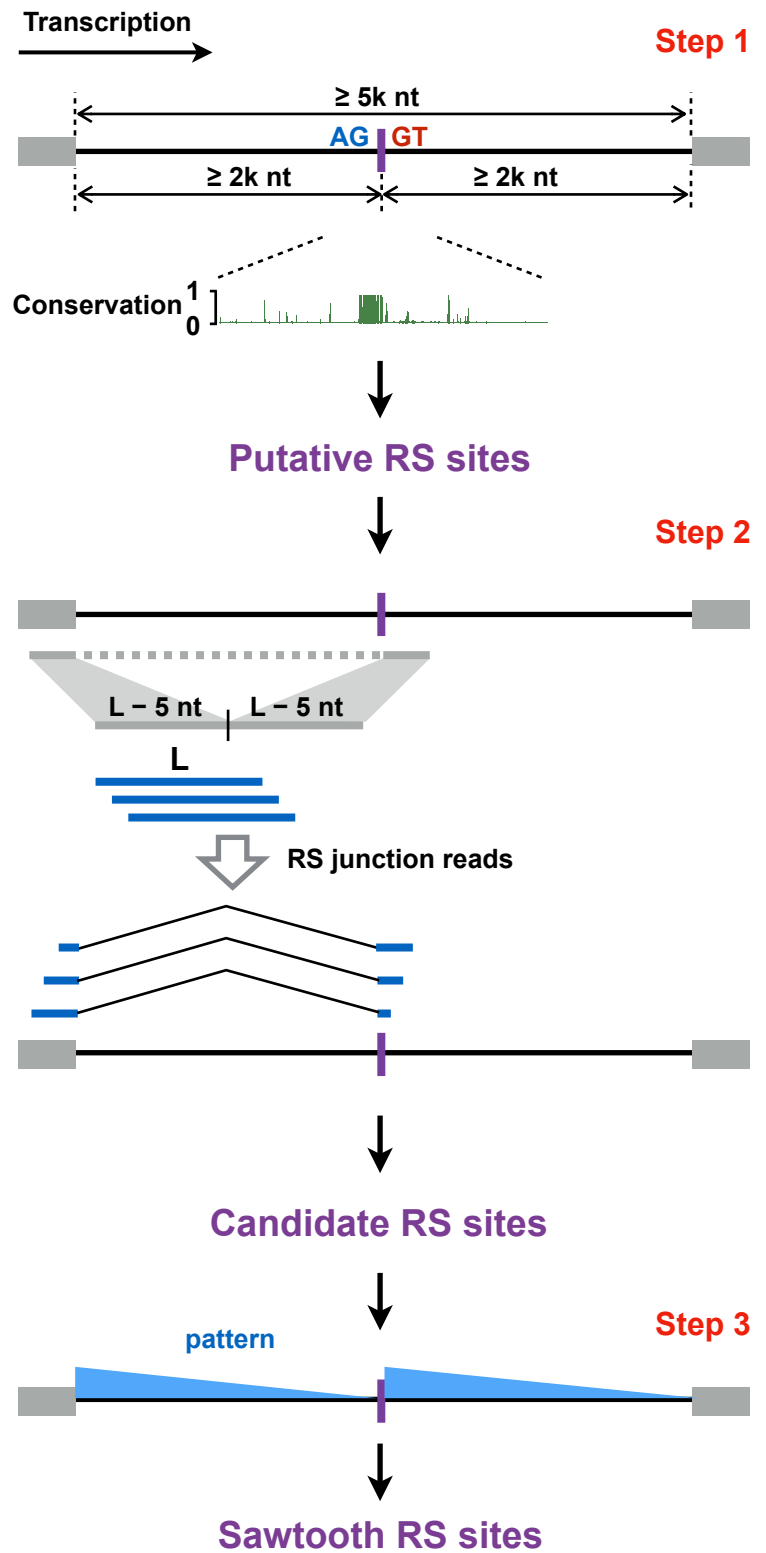

Supplement: S2 Fig — Potential RS sites were first identified according to six genomic features (Step 1), and RS junction reads were then identified using a custom-built junction index (Step 2), resulting in candidate RS sites. For each candidate RS site, the presence of a sawtooth pattern was evaluated with a series of criteria, and sawtooth RS sites with an obvious sawtooth pattern were selected. (PDF) [file pgen.1007579.s002.pdf]

# A

## Sequencing depth

| Cell line | 10 min     | 15 min     | 30 min      | 60 min      | 120 min     | 4 hr        | 16 hr       | Steady state |
|-----------|------------|------------|-------------|-------------|-------------|-------------|-------------|--------------|
| PA1       | 24,064,138 | 53,660,428 | 96,083,184  | 192,488,809 | 260,122,985 | 386,745,248 | 385,816,787 | 125,735,340  |
| H9        | 25,523,929 | 55,747,344 | 89,718,379  | 104,382,001 | 117,792,209 | –           | –           | 80,698,527   |
| FB        | 20,274,512 | 59,032,425 | 130,880,669 | 286,025,212 | 286,511,613 | –           | –           | 85,335,454   |

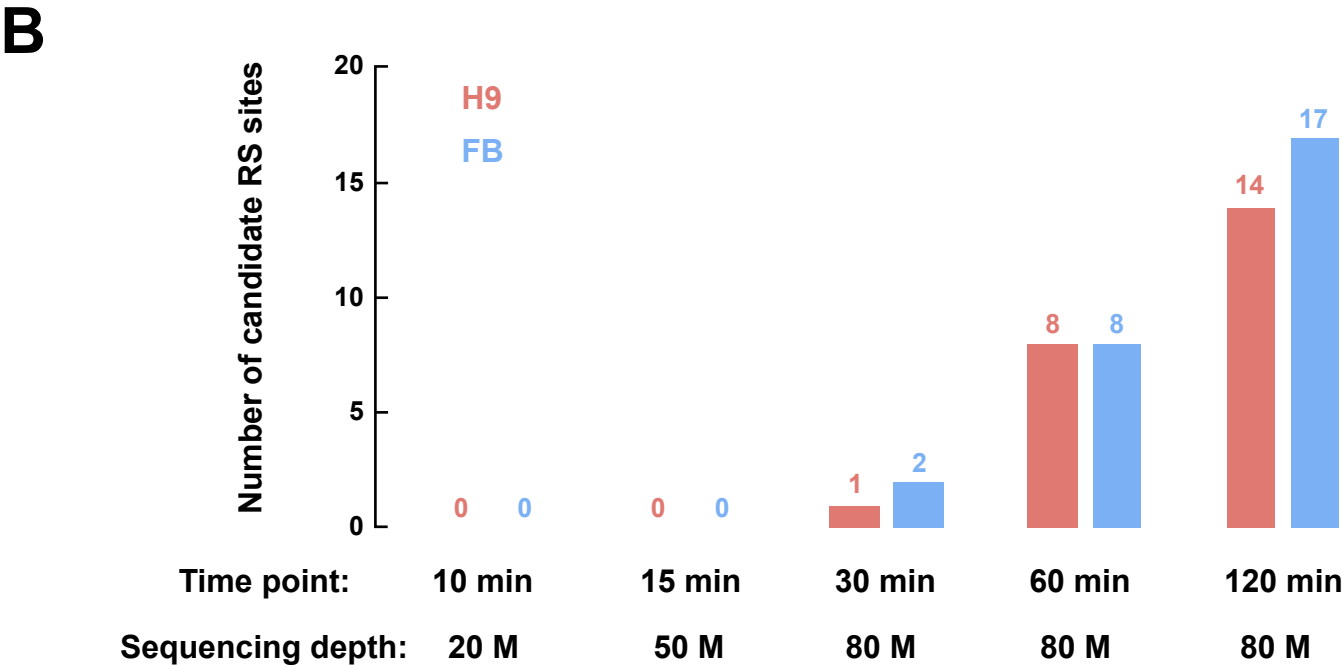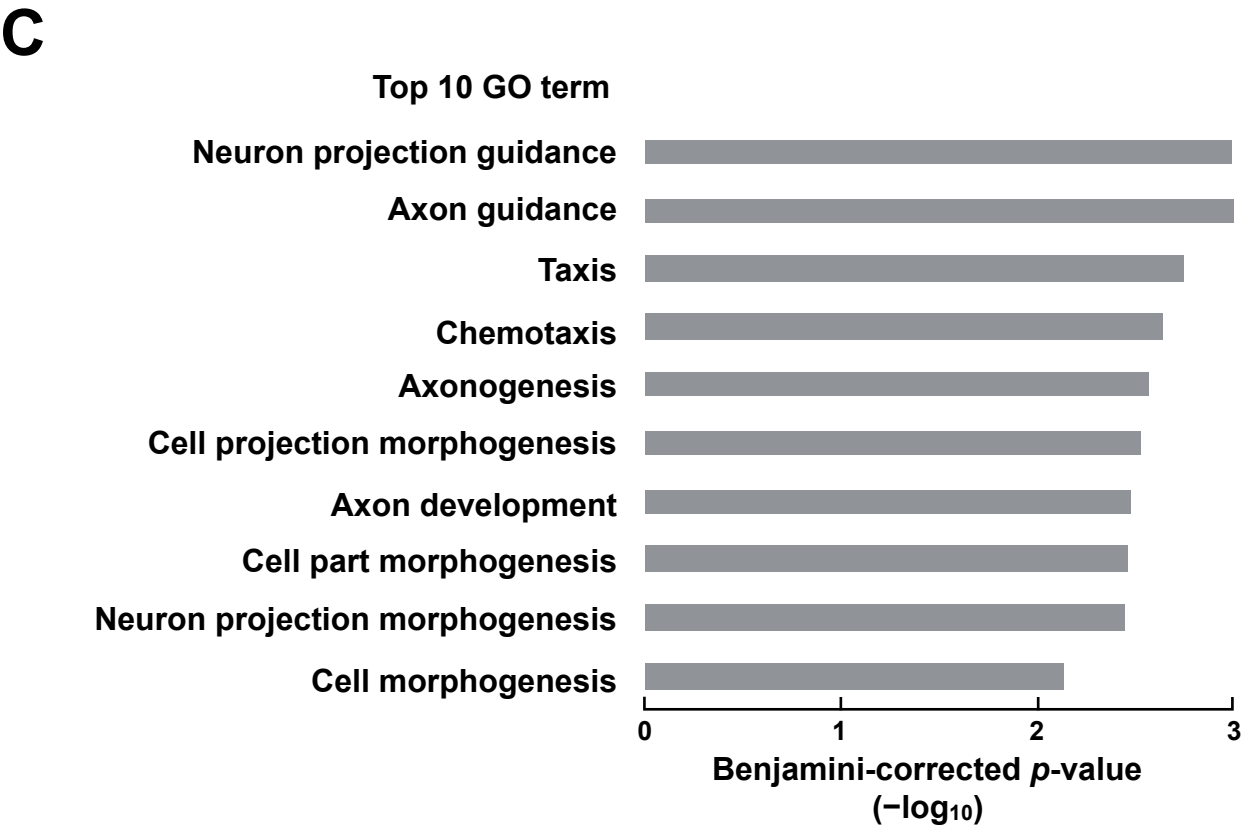

Supplement: S3 Fig — (A) Sequencing depths of 4sUDRB-seq datasets used in this study. (B) More RS sites were identified in FB neurons than H9 cells using the 30 or 120 min 4sUDRB-seq samples at equalized sequencing depth. (C) Genes with recursive splicing are enriched in several gene ontology terms, in particular, neuron projection guidance and axon guidance. (PDF) [file pgen.1007579.s003.pdf]

A

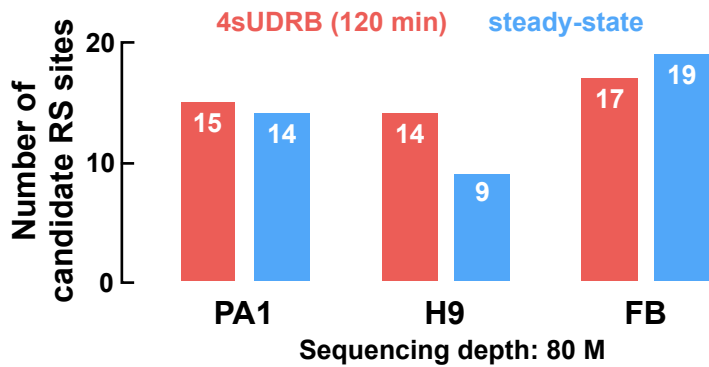

B

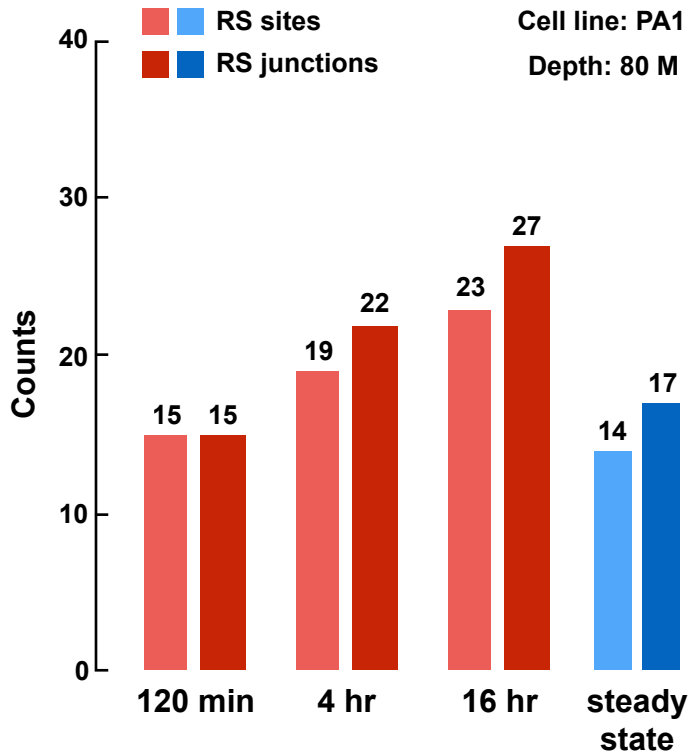

C

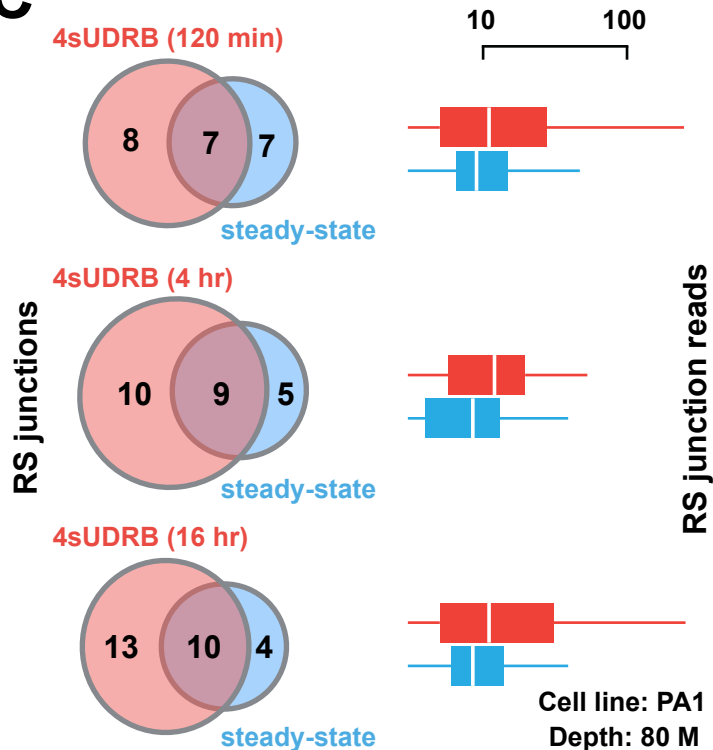

D

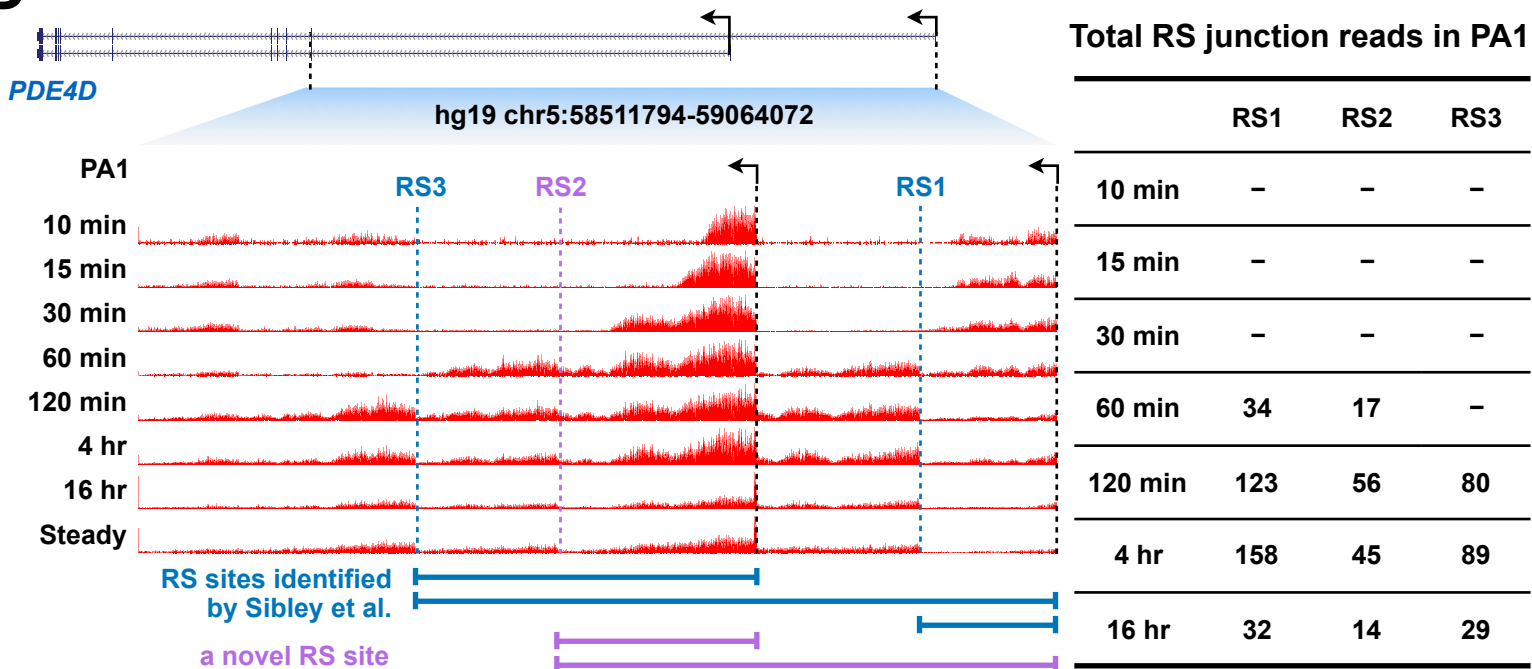

Supplement: S5 Fig — (A) At the same sequencing depth (80 M), more RS sites were identified in the 120 min 4sUDRB-seq datasets (red bars) than the steady-state RNA-seq datasets (blue bars) for PA1 and H9. (B) More RS sites and RS junctions were detected using the 4sUDRB-seq datasets with long 4sU labeling time (red bars) than the steady-state RNA-seq dataset (blue bars) in PA1 cells. (C) Most RS sites identified using the steady-state RNA-seq dataset were also found using 4sUDRB-seq (left Venn diagrams). The RS junctions were supported by comparable numbers of 4sUDRB-seq reads and RNA-seq reads at the same sequencing depth (right bar plots; outliers omitted for clarity). (D) The 4sUDRB-seq signal profiles at the PDE4D gene. The left panel shows three RS sites identified in PA1 cells, including two sites reported by Sibley et al. (RS1 and RS3, blue) and one novel site (RS2, purple). The right panel shows the numbers of RS junction reads supporting the three RS sites in the PA1 4sUDRB-seq data. (PDF) [file pgen.1007579.s005.pdf]

**A**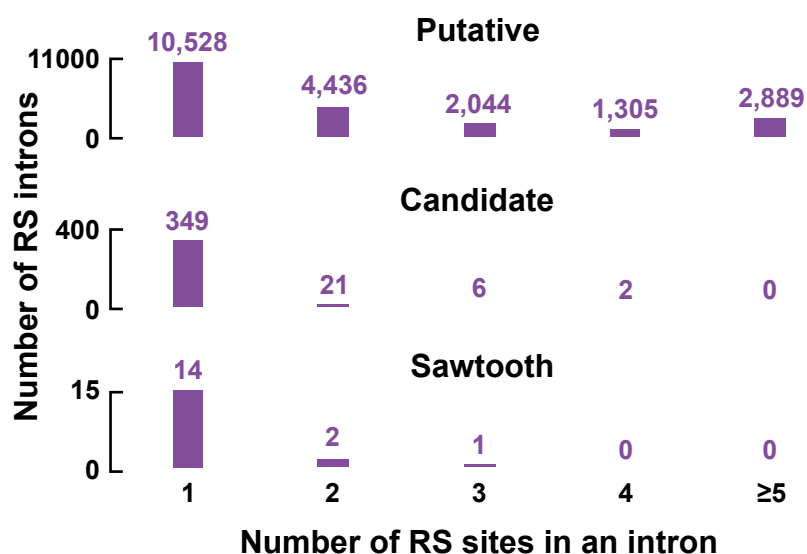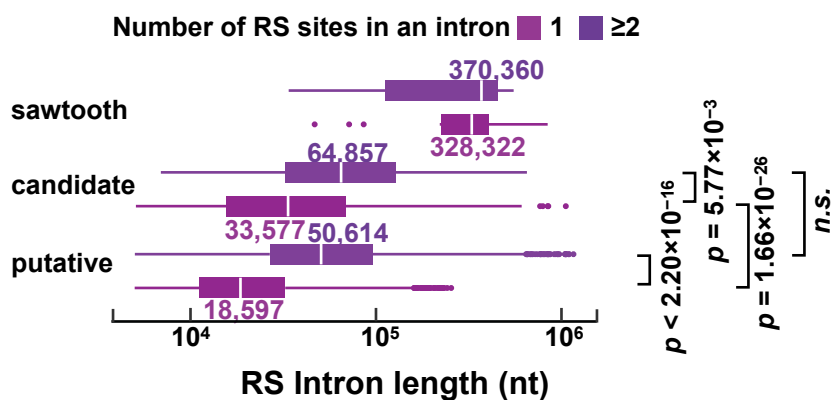**B**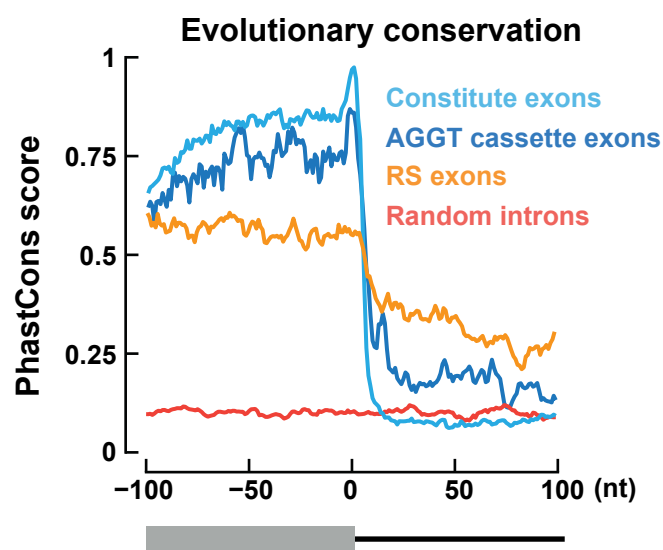**C**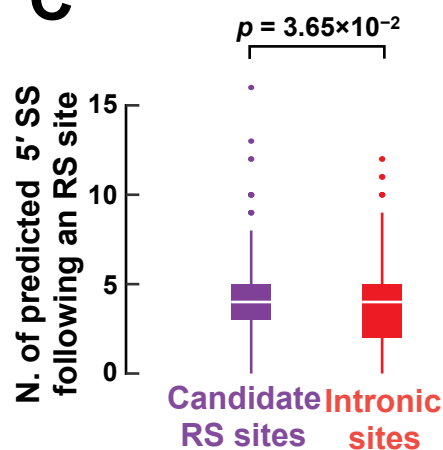**D**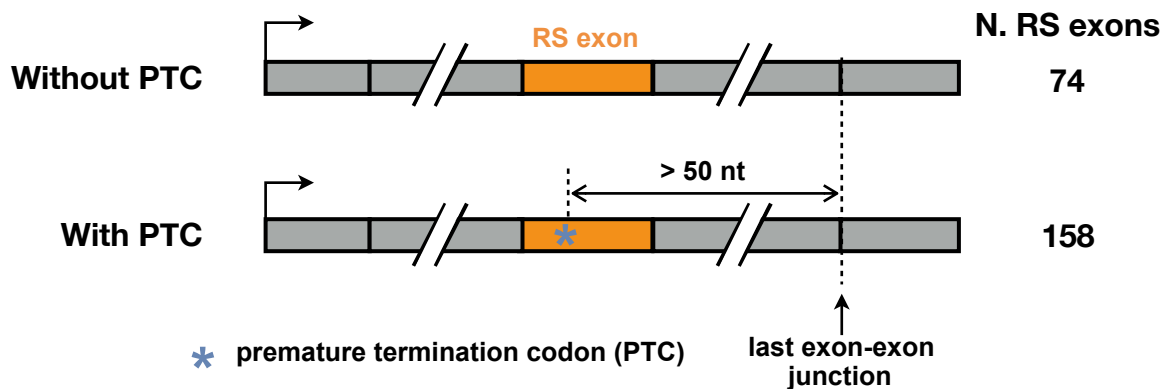

Supplement: S6 Fig — (A) Most RS introns have one RS site (top panel), whereas introns with more than two RS sites are significantly longer than introns with one RS site (bottom panel; medians and Wilcoxon rank-sum test p-values are labeled). (B) The genomic regions around the 5′ splice sites of RS exons (orange) are more evolutionarily conserved than randomly chosen intronic regions (red), albeit not as conserved as constitutive exons (light blue) and cassette exons with the AGGT motif (dark blue). (C) RS sites are followed by more predicted 5′ splice sites than randomly chosen intronic sites are (Wilcoxon rank-sum test). (D) Sixty-eight percent of RS exons contain at least one premature termination codon located more than 50 nucleotides upstream of the last exon-exon junction. (PDF) [file pgen.1007579.s006.pdf]
