# Supplementary material for: The temporal landscape of recursive splicing during Pol II transcription elongation in human cells
Source: PLoS Genet. 2018 Aug 27;14(8):e1007579. doi: 10.1371/journal.pgen.1007579 (PMC6110456; doi:10.1371/journal.pgen.1007579)

A

435 putative RS sites identified in Sibley et al.

Filters	Counts	Percentage
intron length ≥ 5,000 nt	230	53%
high conservation (PhastCon Score ≥ 0.5)	113	26%
not annotated splice sites	429	99%
away from annotated splice site (≥ 2,000 nt)	139	32%
located in annotated introns	292	67%
strong strength as 3′ splice site (score ≥ 5)	395	91%
Filtered RS sites	20	5%

20 filtered RS sites by Sibley et al.

Classified in this study	Putative RS sites	Candidate RS sites	Sawtooth RS sites
Number of RS sites	20	12	9

B

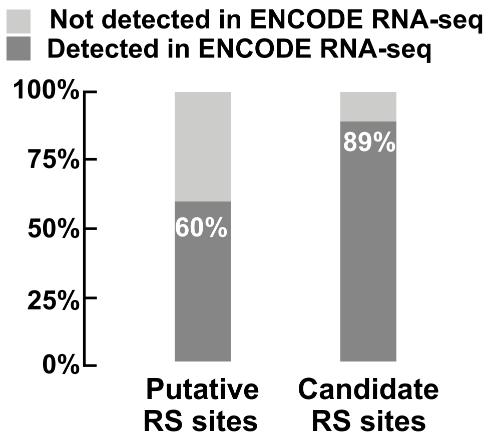

C

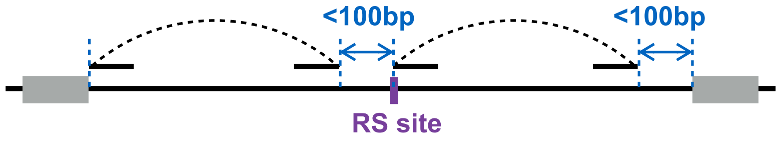

342 candidate RS sites in this study		
lariats	lariat reads	RS sites
23	266	15

230 putative RS sites in introns (≥ 5,000 nt) by Sibley et al.		
lariats	lariat reads	RS sites
15	153	12

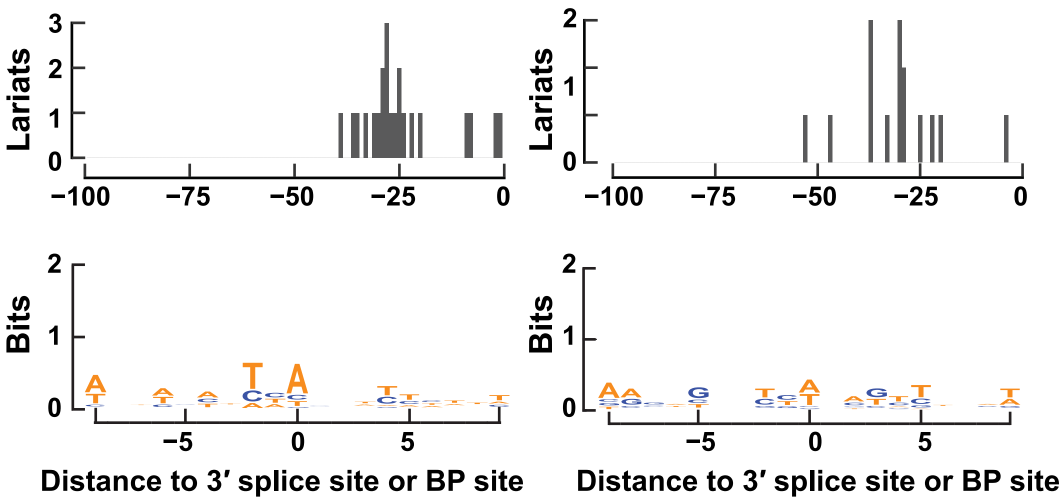

D

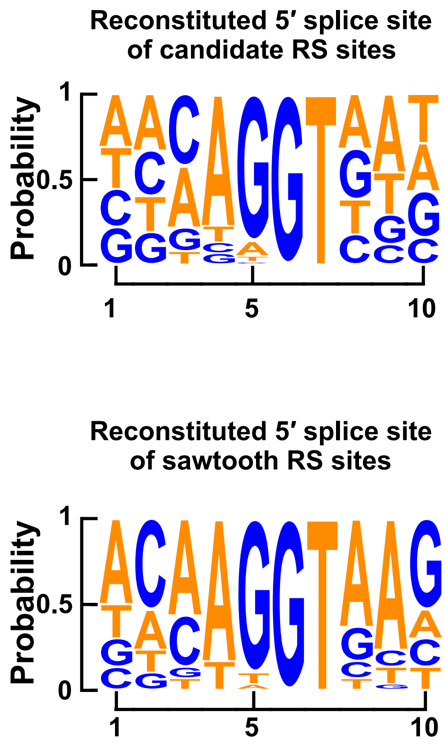

Supplement: S4 Fig — (A) Comparison between RS sites identified by previous study [4] and this study. We note that 12 of the 20 filtered RS sites (top panel) could be detected by our pipeline (bottom panel). (B) Sixty percent of putative RS sites and 89% of candidate RS sites could be detected with recursive splicing junctions using ENCODE total RNA-seq samples. (C) Identification of recursive splicing intron lariats to support the occurrence of recursive splicing (top panel, Materials and methods). The branch point identified by lariat reads showed proper distance to 3′ splice site (middle panel) and canonical sequence motif (bottom panel). (D) The sequence motifs of reconstituted 5′ splice site of candidate (top panel) and sawtooth (bottom panel) RS sites. (PDF) [file pgen.1007579.s004.pdf]
