# Supplementary material for: The temporal landscape of recursive splicing during Pol II transcription elongation in human cells
Source: PLoS Genet. 2018 Aug 27;14(8):e1007579. doi: 10.1371/journal.pgen.1007579 (PMC6110456; doi:10.1371/journal.pgen.1007579)

A

Time point of RS onset	Post-transcriptional	Undetermined	Co-transcritpional
	Time needed by Pol II to transcribe the entire gene		
10 min	0	0~10	>10
15 min	<10	10~15	>15
30 min	<15	15~30	>30
60 min	<30	30~60	>60
120 min	<60	60~120	>120
240 min (4 hr)	<120	120~240	>240
960 min (16 hr)	<240	240~960	>960

B

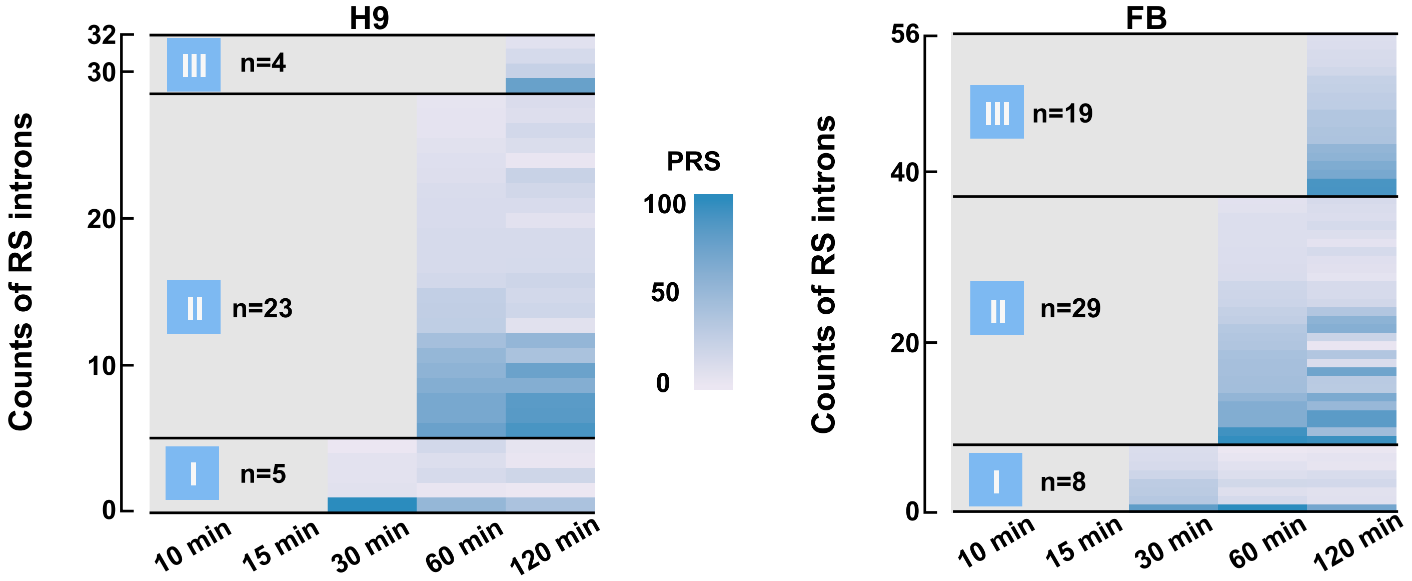

	H9 Post-transcriptional	H9 Undetermined	H9 Co-transcriptional	FB Post-transcriptional	FB Undetermined	FB Co-transcriptional
III	2 (50%)	0 (0%)	2 (50%)	6 (31%)	3 (16%)	10 (53%)
II	11 (48%)	9 (39%)	3 (13%)	12 (41%)	10 (35%)	7 (24%)
I	1 (20%)	1 (20%)	3 (60%)	2 (25%)	1 (12%)	5 (63%)

C

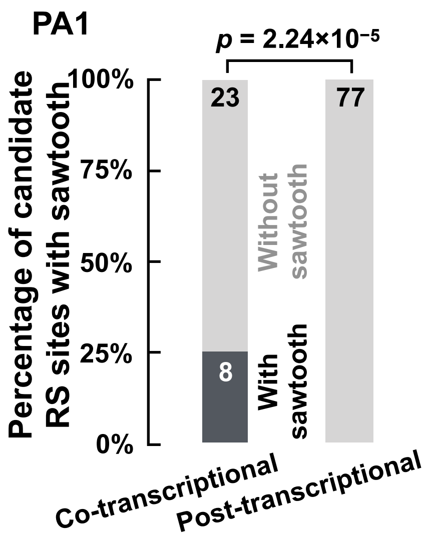

D

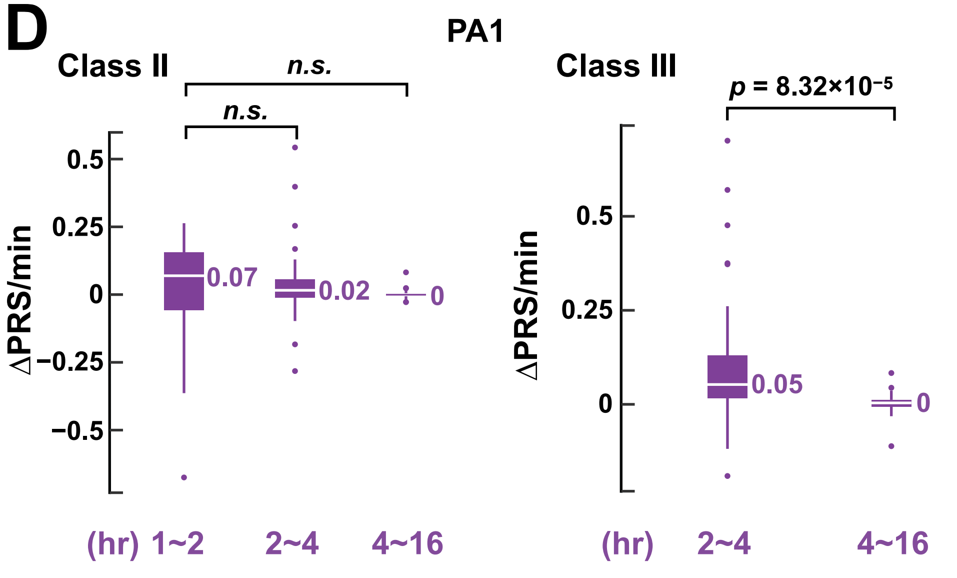

Supplement: S7 Fig — (A) The criteria for defining post- and co-transcriptional recursive splicing. (B) The time courses of recursive splicing in H9 cells and FB neurons. (C) Among candidate RS sites, a higher percentage of co-transcriptional sites shows the sawtooth pattern than post-transcriptional sites (26% vs. 0%, p-value = 2.24×10^−5^, Fisher’s exact test). (D) PRS drops rapidly after the onset of recursive splicing (Wilcoxon rank-sum test). (PDF) [file pgen.1007579.s007.pdf]
