# Supplementary material for: The temporal landscape of recursive splicing during Pol II transcription elongation in human cells
Source: PLoS Genet. 2018 Aug 27;14(8):e1007579. doi: 10.1371/journal.pgen.1007579 (PMC6110456; doi:10.1371/journal.pgen.1007579)
Supplement: S8 Fig — (A) The Pol II elongation rates of genes with RS introns (RS genes) are higher than those of non-RS genes with or without long introns in FB neurons (left panel), PA1 cells (middle panel), and H9 (right panel) cells. Medians and Wilcoxon rank-sum test p-values are labeled. (B) RS genes are enriched in several histone marks. Compared with non-RS genes (black lines), RS genes (purple line) have a higher density of histone modification (left panel: H3K79me2, middle panel: H4K20me1, right panel: H3K4me1) near the transcription start site (TSS). (C) There is no significant difference in H3K36me3 levels between RS genes (purple line) and non-RS genes (black line). (D) RS genes in FB neurons, PA1, and H9 cells show a series of genomic features that are correlated with fast Pol II elongation. (PDF) [file pgen.1007579.s008.pdf]

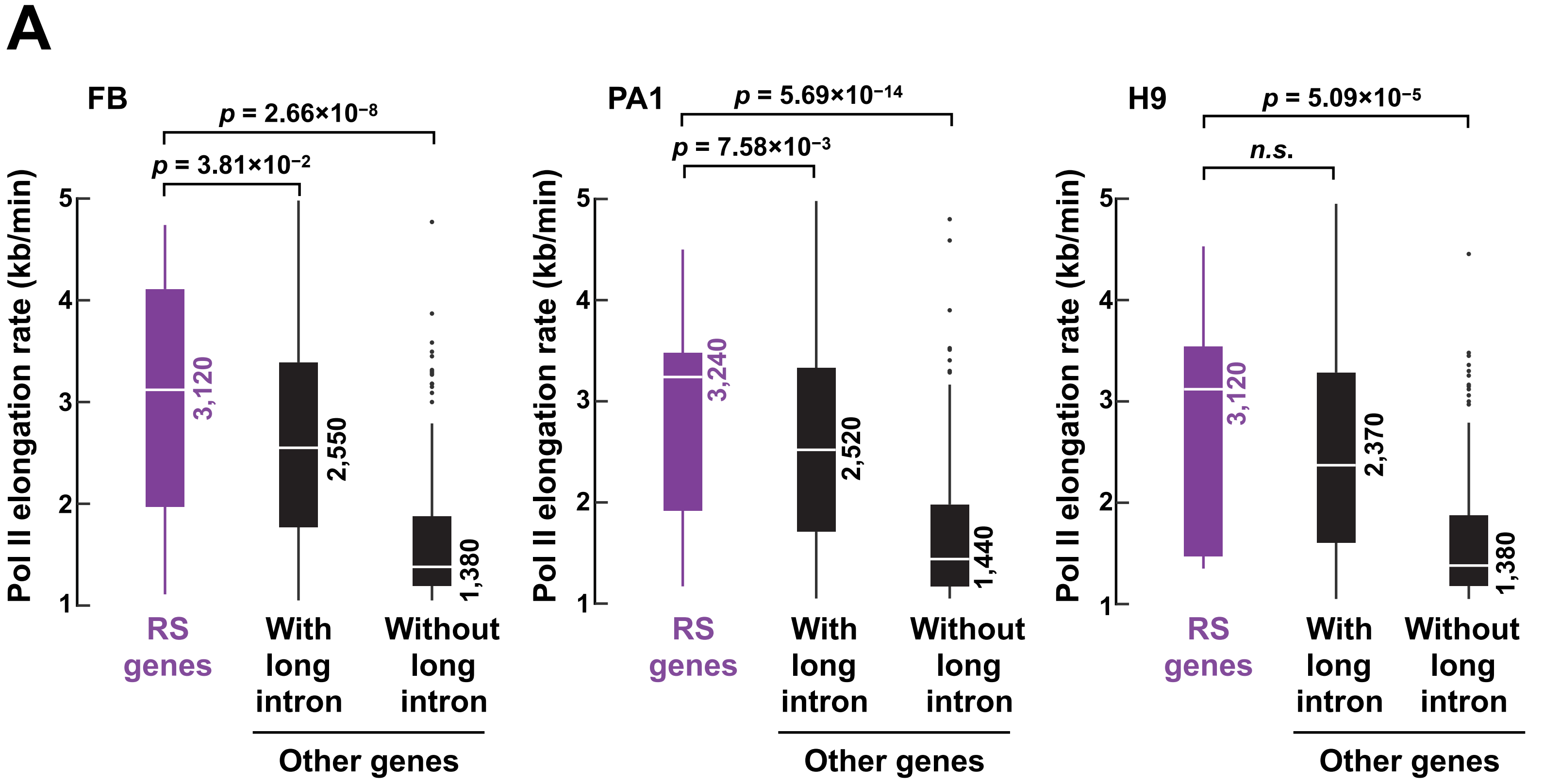

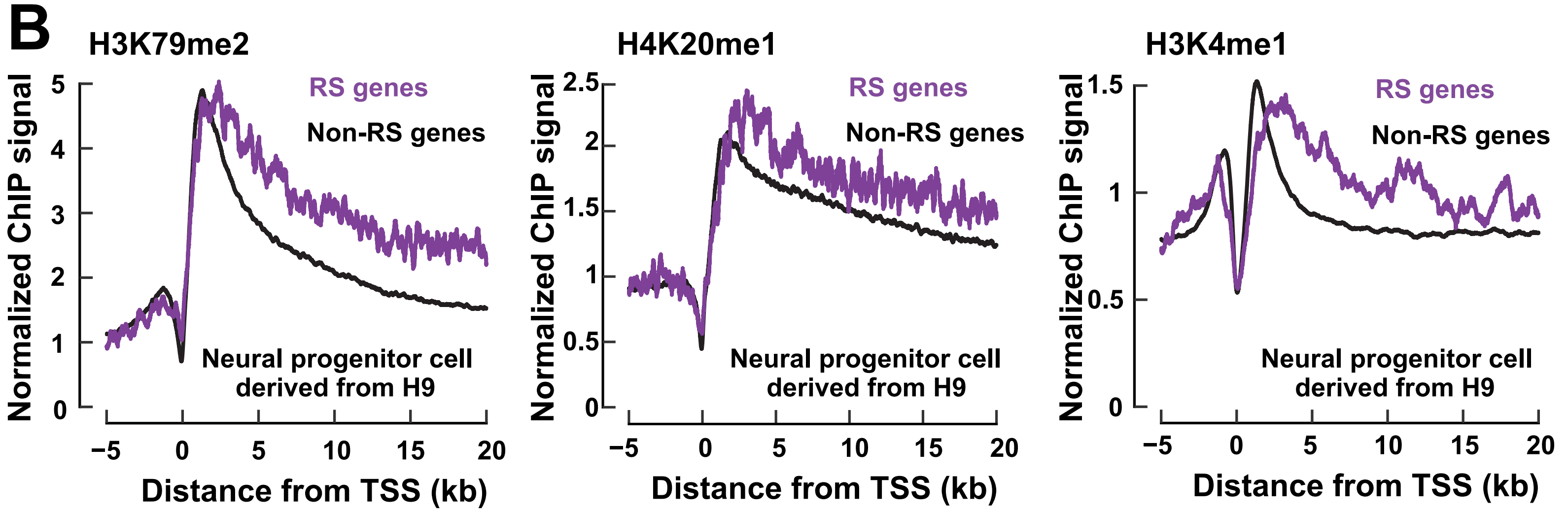

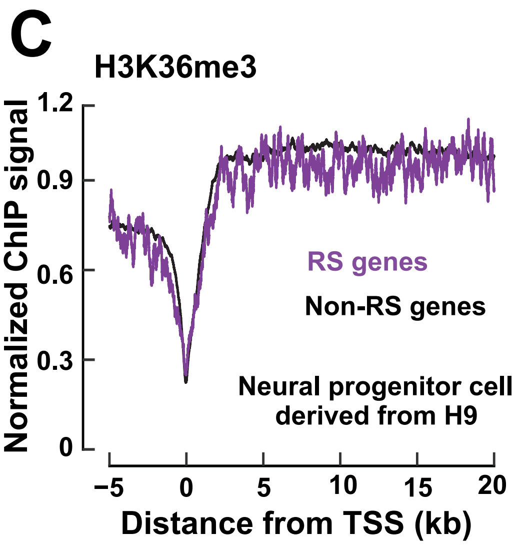

D

Feature	FB	PA1	H9
Length of the first intron	↗ (p = 1.58×10^{-2})	↗ (p = 1.47×10^{-10})	n. s.
Exon density	↘ (p = 1.02×10^{-10})	↘ (p = 1.45×10^{-18})	↘ (p = 3.13×10^{-3})
Simple repeats	↗ (p = 1.14×10^{-2})	↗ (p = 4.02×10^{-4})	↗ (p = 1.57×10^{-2})
GC content	↘ (p = 5.17×10^{-3})	n. s.	↘ (p = 2.93×10^{-2})

↗: RS genes>Non-RS genes; ↘: RS genes<Non-RS genes; n. s.: not statistically significant
