# Supplementary material for: The temporal landscape of recursive splicing during Pol II transcription elongation in human cells
Source: PLoS Genet. 2018 Aug 27;14(8):e1007579. doi: 10.1371/journal.pgen.1007579 (PMC6110456; doi:10.1371/journal.pgen.1007579)
Supplement: S9 Fig — (A) A comparison of the occurrence of RS junction reads (R1) and canonical junction reads (R2) in H9 cells (left) and FB neurons (right). (B) Number of reads that support RS lariats or full-length lariats in RS introns. (PDF) [file pgen.1007579.s009.pdf]

A

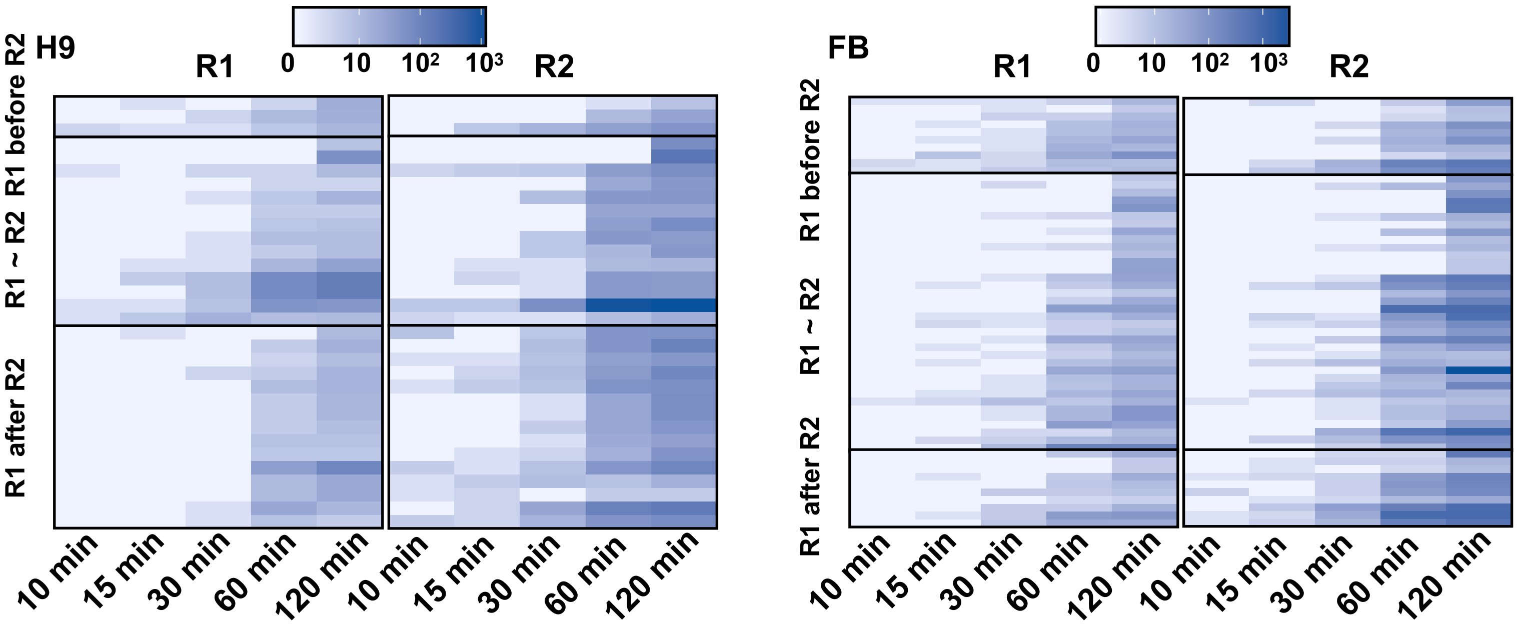

	R1 occurs before R2 (R1 before R2)	R1 and R2 at the same time point (R1 ~ R2)	R1 occurs after R2 (R1 after R2)
H9	3 (9%)	14 (44%)	15 (47%)

	R1 occurs before R2 (R1 before R2)	R1 and R2 at the same time point (R1 ~ R2)	R1 occurs after R2 (R1 after R2)
FB	10 (18%)	36 (64%)	10 (18%)

B

327 RS introns in PA1	With full length lariats in PA1	Without full length lariats in PA1	With full length lariats in other cells	Without full length lariats in other cells
With recursive lariats in PA1	0	9	1	8
Without recursive lariats in PA1	12	306	33	285

82 RS introns in H9	With full length lariats in H9	Without full length lariats in H9	With full length lariats in other cells	Without full length lariats in other cells
With recursive lariats in H9	1	1	1	1
Without recursive lariats in H9	2	78	8	72

129 RS introns in FB	With full length lariats in FB	Without full length lariats in FB	With full length lariats in other cells	Without full length lariats in other cells
With recursive lariats in FB	0	0	0	0
Without recursive lariats in FB	5	124	15	114
